# Diversity and Strain Specificity of Plant Cell Wall Degrading Enzymes Revealed by the Draft Genome of *Ruminococcus flavefaciens* FD-1

**DOI:** 10.1371/journal.pone.0006650

**Published:** 2009-08-14

**Authors:** Margret E. Berg Miller, Dionysios A. Antonopoulos, Marco T. Rincon, Mark Band, Albert Bari, Tatsiana Akraiko, Alvaro Hernandez, Jyothi Thimmapuram, Bernard Henrissat, Pedro M. Coutinho, Ilya Borovok, Sadanari Jindou, Raphael Lamed, Harry J. Flint, Edward A. Bayer, Bryan A. White

**Affiliations:** 1 Department of Animal Sciences, University of Illinois at Urbana-Champaign, Urbana, Illinois, United States of America; 2 Institute for Genomic Biology, University of Illinois at Urbana-Champaign, Urbana, Illinois, United States of America; 3 Microbial Ecology Group, Rowett Institute of Nutrition and Health, University of Aberdeen, Aberdeen, United Kingdom; 4 Architecture et Fonction des Macromolécules Biologiques, CNRS and Universités Aix-Marseille I & II, Marseille, France; 5 Department of Molecular Microbiology and Biotechnology, Tel Aviv University, Ramat Aviv, Israel; 6 Department of Biological Chemistry, The Weizmann Institute of Science, Rehovot, Israel; University of Hyderabad, India

## Abstract

**Background:**

*Ruminococcus flavefaciens* is a predominant cellulolytic rumen bacterium, which forms a multi-enzyme cellulosome complex that could play an integral role in the ability of this bacterium to degrade plant cell wall polysaccharides. Identifying the major enzyme types involved in plant cell wall degradation is essential for gaining a better understanding of the cellulolytic capabilities of this organism as well as highlighting potential enzymes for application in improvement of livestock nutrition and for conversion of cellulosic biomass to liquid fuels.

**Methodology/Principal Findings:**

The *R. flavefaciens* FD-1 genome was sequenced to 29x-coverage, based on pulsed-field gel electrophoresis estimates (4.4 Mb), and assembled into 119 contigs providing 4,576,399 bp of unique sequence. As much as 87.1% of the genome encodes ORFs, tRNA, rRNAs, or repeats. The GC content was calculated at 45%. A total of 4,339 ORFs was detected with an average gene length of 918 bp. The cellulosome model for *R. flavefaciens* was further refined by sequence analysis, with at least 225 dockerin-containing ORFs, including previously characterized cohesin-containing scaffoldin molecules. These dockerin-containing ORFs encode a variety of catalytic modules including glycoside hydrolases (GHs), polysaccharide lyases, and carbohydrate esterases. Additionally, 56 ORFs encode proteins that contain carbohydrate-binding modules (CBMs). Functional microarray analysis of the genome revealed that 56 of the cellulosome-associated ORFs were up-regulated, 14 were down-regulated, 135 were unaffected, when *R. flavefaciens* FD-1 was grown on cellulose versus cellobiose. Three multi-modular xylanases (ORF01222, ORF03896, and ORF01315) exhibited the highest levels of up-regulation.

**Conclusions/Significance:**

The genomic evidence indicates that *R. flavefaciens* FD-1 has the largest known number of fiber-degrading enzymes likely to be arranged in a cellulosome architecture. Functional analysis of the genome has revealed that the growth substrate drives expression of enzymes predicted to be involved in carbohydrate metabolism as well as expression and assembly of key cellulosomal enzyme components.

## Introduction

Ruminococci are cellulolytic Gram-positive cocci in the order ‘Clostridiales’, which inhabit the rumen community. They are responsible for degrading cellulosic plant cell wall material, and also for solubilizing components that can be utilized by other rumen bacteria [1]. Members of the *Ruminococcus* genus were first described by A. K. Sijpesteijn in the early part of the twentieth century which were followed by equivalent descriptions by R. E. Hungate [2], [3]. The *R. flavefaciens* FD-1 strain was first isolated by Marvin P. Bryant from a bolus containing ruminal microorganisms used to improve rumen function in calves [4]. Although the *R. flavefaciens* type strain is C94, its cellulolytic activity is much lower than that of FD-1, particularly on more crystalline forms of cellulose [5]. *R. flavefaciens* strains are known to vary widely in their activities against intact plant cell wall material, and against different forms of cellulose, but many strains share with FD-1 the ability to attack highly crystalline forms of cellulose [6]. Most *R. flavefaciens* strains exhibit a preference for more complex sugars, as evidenced by the uptake of cellobiose but the absence of an uptake system for glucose [7]. *R. flavefaciens*-related bacteria are also thought to play a role in plant cell wall polysaccharide digestion in the large intestine in herbivorous mammals and in man [8].

The diversity and organization of cellulases and other proteins involved in plant cell wall breakdown by rumen cellulolytic bacteria is fundamental to understanding how ruminants extract energy from their diet. The cellulolytic enzyme system from *R. flavefaciens* FD-1 has been shown to include a variety of exo-β-1,4-glucanases, endo-β-1,4-glucanases and cellodextrinases [9], [10], [11], [12]. Difficulties were encountered in initial fractionation of these enzymes as they appeared to exist in high-molecular-weight protein complexes resembling cellulosomes [12], [13], and enzymatic activity was lost rapidly when the complexes were disrupted [12]. Individual β-glucanase genes (*celA*, *celB*, *celC*, and *celD*) were cloned from *R. flavefaciens* FD-1 with a view to studying their regulation [14], [15], [16], [17], [18]. Meanwhile, parallel studies in the related *R. flavefaciens* strain 17 also led to the sequence analysis of a number of xylanases and cellulases. This revealed the presence of multiple catalytic modules in xylanases [19], [20], [21] and the presence of non-catalytic dockerins [19], [22] and of substrate-binding modules [23] in both cellulases and xylanases. The hypothesis that these dockerin-containing enzymes are organized into cellulosomes was supported by the discovery of the *sca* cluster of genes in *R. flavefaciens* 17 that encodes the cohesin-containing scaffolding or anchoring proteins ScaA, B, C and E [24], [25], [26], [27]. Evidence was obtained in *R. flavefaciens* 17 that many enzymes are assembled into the cellulosome complex via cohesin-dockerin interactions involving the ScaA “scaffoldin” protein, while other, currently unknown, proteins appear to be accommodated via the ScaC adaptor protein [24], [27]. ScaA in turn binds via its C-terminal dockerin to ScaB, which is held into the cell surface via another cohesin-dockerin interaction with the cell-wall anchored protein ScaE [25], [26]. The homologous *sca* cluster has now been identified in *R. flavefaciens* FD-1 and shows close alignment in gene order with that in *R. flavefaciens* 17, although interesting interstrain differences exist in the modular structures of ScaA and ScaB [28]. Experimental verification of specific cohesin-dockerin interactions indicates that a broadly similar cellulosome organization exists in *R. flavefaciens* FD-1 and 17 [28]. Genes encoding several molecular chaperones (*groES*, *groEL*, and *dnaK*) have also been described from *R. flavefaciens* FD-1 that could be involved in the assembly of cellulosome-like structures [29].

Genome sequencing of *R. flavefaciens* FD-1 offers the prospect of obtaining far more extensive information on the range and diversity of enzymatic and structural components of the cellulosome, on its organization, range of cohesin-dockerin interactions, and on the regulation and assembly of cellulosomal subunits. At the same time, significant information is obtained on non-cellulosomal proteins. Here, the genome of *R. flavefaciens* FD-1 was sequenced to approximately 29×-coverage, and the resulting collection of contiguous sequences screened for open reading frames (ORFs) that may encode proteins involved in fiber-degradation. The large number of protein-encoding sequences containing dockerin modules detected indicates that *R. flavefaciens* FD-1 has the largest collection of cellulosome-associated proteins of any known fiber-degrading bacterium thus far described. Comparison with known enzymes from *R. flavefaciens* 17 indicates many subtle differences between the two strains in modular organization among enzymes involved in lignocellulose degradation. Additionally, gene expression profiling using microarray technology has allowed us to obtain functional information about the majority of the genome by comparing gene expression when *R. flavefaciens* FD-1 is grown on cellulose or cellobiose. These experiments have revealed that the substrate drives expression of the different enzymes involved in the degradation of cellulosic material, and suggests that the cellulosome plays a central role in this process.

## Results and Discussion

### Assessing functional coverage of the *R. flavefaciens* FD-1 draft genome

In combination with suppressive subtractive hybridization (SSH) sequences obtained from our previous comparative studies of *R. flavefaciens* FD-1 and JM1 [30], 430,226 sequence reads from GS FLX pyrosequencing and 28,681 ESTs from Sanger sequencing were assembled using the PHRED/PHRAP system [31], [32], [33], producing 119 primary contiguous sequences (Table 1). These contigs range in size from 205 bp (i.e. single unique reads) to 31,187 bp. A total of 4,339 ORFs were identified in *R. flavefaciens* FD-1. Of these, 2,289 (52.8%) could be assigned to biological role categories, 385 (8.9%) were conserved hypothetical proteins or conserved modular proteins, 422 (9.7%) were of unknown function, 79 (1.8%) were unclassified with no assigned role category, and 1,241 (28.6%) encoded hypothetical proteins. There appears to be one ribosomal operon harboring single copies of genes encoding the 16S and 23S rRNA molecules.

**Table 1:** Summary of genome characteristics and features for *Ruminococcus flavefaciens* FD-1.

Molecule length	4,576,399 bp
GC content (%)	45
Total number of open reading frames	4,339
assigned function	2,289 (52.8%)
conserved hypothetical	385 (8.9%)
unknown function	422 (9.7%)
unclassified, no assigned role category	79 (1.8%)
hypothetical proteins	1241 (28.6%)
Average gene length (base pairs)	918
Transfer RNA	56
Ribosomal RNA	7
ncRNA	2
Ribozyme	1
tmRNA	1
Percent coding (%)	87.1
Percent coding or tRNA, rRNA, or repeat (%)	87.1

The total amount of unique sequence is 4,573,803 bp with an average GC content of 45%. This compares with an approximate estimate of 4.4 Mb genome based on pulsed-field gel estimates for the genome of the closely related strain, *R. flavefaciens* 17. According to the Poisson distribution, 29× coverage worth of genome sequencing should produce approximately 99.999% of the genome. An inventory of functional sequences was conducted based on TIGR's Annotation Engine output. It was decided to focus first on sequences related to amino acid biosynthesis for which we expected relatively conserved biosynthetic pathways. Previously, a gapped genomic approach was used to provide a functional analysis of amino acid metabolism of *Thiobacillus ferrooxidans* and to estimate the extent of genome coverage [34]. Rumen bacteria, such as *R. flavefaciens*, have long been documented to require free ammonia in the medium, therefore the organism must be able to synthesize all of the necessary amino acids *de novo* [35], [36]. MetaCyc was utilized to help visualize the metabolic steps for each of the amino acid families [37].

Of the 90 expected ORFs necessary for biosynthesis of the major families of amino acids in *R. flavefaciens* FD-1, 83 were detected suggesting that the overall genome size predicted by PFGE may be an underestimate. Nineteen ORFs encode enzymes involved in the biosynthesis of aromatic amino acids, and 19 ORFs encode enzymes involved in biosynthesis of the aspartate family of amino acids. In addition 23 ORFs were involved with the biosynthesis of the glutamate family, 19 ORFs with the pyruvate family, eight ORFs with the serine family and ten ORFs with the histidine family of amino acids (Table S1).

An inventory of full and partial ORFs revealed several sequences that matched those obtained previously by cloning and sequencing of individual genes. These include genes implicated in cellulose degradation (*celA*, *celB* and *celD*), as well as ammonia assimilation, and the heat shock (general stressor) response. This genome assembly has corrected for sequencing errors in the *celB* sequence currently in GenBank (gi|736356). Genome sequencing showed that the *celB* gene is actually 3471 bp, and has homology with the *R. flavefaciens* 17 family 44 cellulase. New features of the *celB* protein are a CBM, T-rich linker region and a dockerin domain. No sequence was detected for *celE* (gi|152634). Sequences matching the previously sequenced glutamate dehydrogenase (*gdhA*; gi|27461937) and glutamine synthetase type III (*glnA*; gi|2895903) were also represented in the draft sequence data: ORF01204 and ORF03347, respectively. Several ORFs that were identified match the cloned heat shock genes: ORF01108 and ORF02365 (for *dnaK*; gi|37779192) and ORF03100 and ORF03101 (for *groESL*; gi|37779196).

### Cellulases and associated glycoside hydrolases

**Figure 1:**
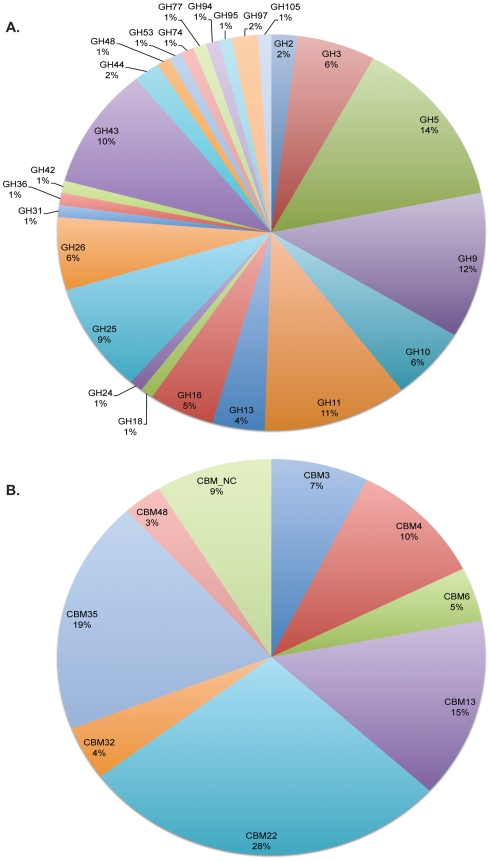
Abundance of glycoside hydrolase modules and carbohydrate-binding modules detected in *R. flavefaciens* FD-1. A. The 101 GH family modules predicted in *R. flavefaciens* FD-1. B. The 68 detected CBMs, according to family type.

Based on comparison with the Carbohydrate Active Enzymes (CAZy) database (http://www.cazy.org) [38], sequences from the *R. flavefaciens* FD-1 genome were classified according to families and modules. Glycoside hydrolases, including those found in cellulases and hemicellulases (the latter referred to as xylanases and mannanases), have been organized into 114 families in the CAZy database. ORFs containing at least one predicted GH module can be seen in Table S2. The distribution of the 25 glycoside hydrolase families identified in *R. flavefaciens* FD-1 is dominated by families 5 and 9 (14 and 12 identified catalytic modules, respectively; Figure 1). These GH modules are characteristic of processive, endo-acting beta-1,4-glucanases. The repertoire of detected GH modules is summarized in Figure 1A and, in addition to families 5 and 9, includes representatives of families 2, 3, 10, 11, 13, 16, 18, 24, 25, 26, 31, 36, 42, 43, 44, 48, 53, 74, 77, 94, 95, 97, and 105. The presence of a GH family 48 module in ORF03925 is indicative of the presence of a processive exo-acting beta-1,4-glucanase. This ORF is also phylogenetically related to Cel48A from *R. albus* [39], which provides further evidence that this enzyme is a processive exo-acting enzyme (Figure S1; Table S3). A dockerin has also been detected in the same ORF supporting its integration into the *R. flavefaciens* FD-1 cellulosome.

### Genes associated with the breakdown and utilization of xylans

**Figure 2:**
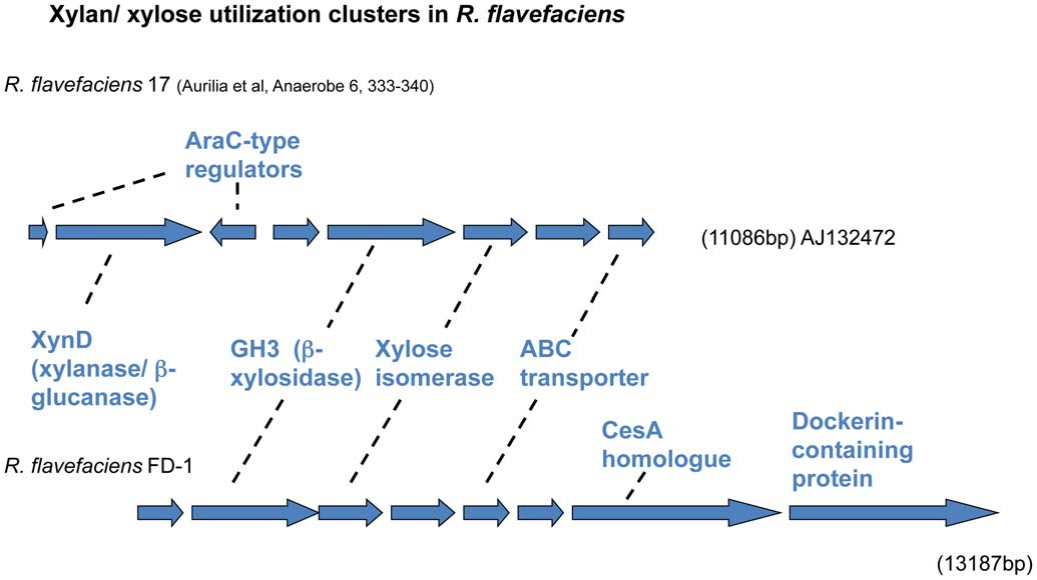
Comparison of chromosomal regions encoding xylose isomerase and associated genes involved in utilization of xylo-oligosaccharides between *R. flavefaciens* strains FD-1 and 17.

One of the GH family 3 modules found in ORF02396 is homologous with the GH3 module from a β-xylosidase gene, which is included in a xylan utilization operon previously identified in *R. flavefaciens* 17 [40]. This GH3 enzyme is presumed to function as a β-xylosidase and/or α-arabinofuranosidase, since these activities were associated with the cloned region [41]. Homology extends downstream to include the gene for xylose isomerase (*xsi*), and three genes encoding components of an ABC transporter system (*ugpA*, *B* and *E*) (Figure 2). The gene encoding xylulokinase is located elsewhere in the FD-1 genome (ORF02846) whereas in most bacteria it is adjacent to the isomerase gene. ORF02390 encoding a dockerin-containing protein is found immediately downstream of the transporter genes in FD-1, while the gene for another dockerin-containing protein, XynD [20], is encoded by the region upstream of the GH3 xylosidase in *R. flavefaciens* 17 (Figure 2).

**Figure 3:**
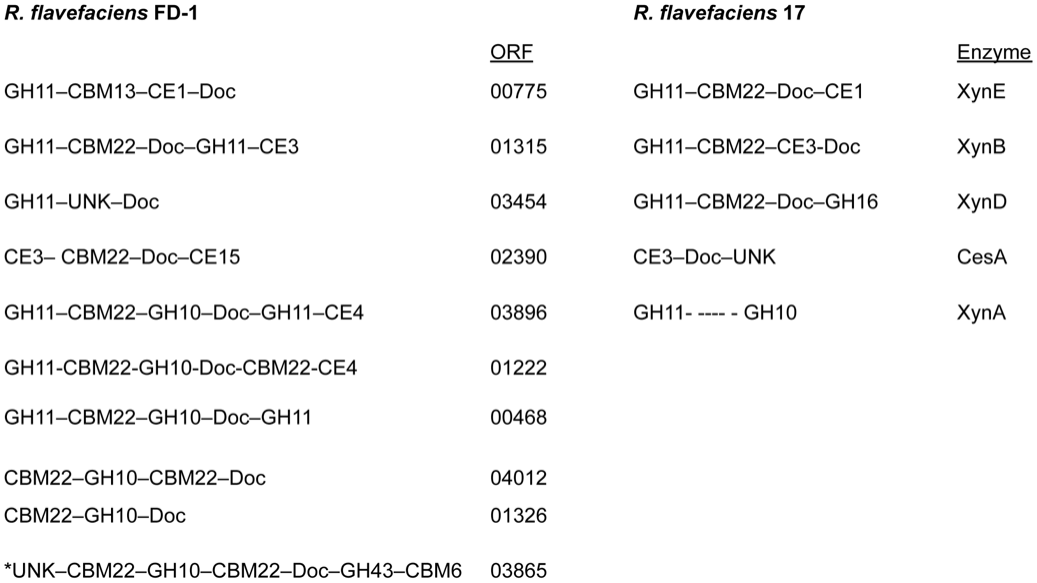
Modular structures of multi-modular enzymes involved in xylan breakdown from *R. flavefaciens* FD-1 and 17. Catalytic modules are indicated by glycoside hydrolase enzyme family (GH10, GH11, CE3 etc). Families of carbohydrate binding modules (CBM22 etc) and dockerin modules (Doc) are also indicated. All complete ORFs carry a predicted signal peptide at the N terminus (not shown). Incomplete ORFs are indicated by an asterisk.

**Figure 4:**
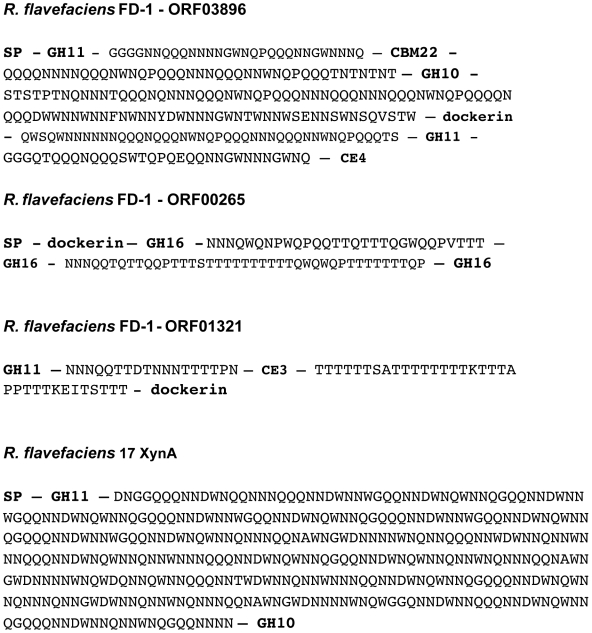
Instances of unusual NQ-rich linker regions in enzymes from *R. flavefaciens* FD-1 and 17. The linker sequences are shown in full, while the catalytic modules and binding modules that they connect are indicated by appropriate abbreviations (GH10 etc).

ORFs that include GH10 or GH11 xylanase modules commonly showed multiple catalytic modules. In one case, GH modules representing family 10 and 43 are detected in the same ORF (ORF03865; Table S2). One larger ORF (ORF03896; 4.5 kb) appears to encode a tetrafunctional endo-1,4-β-xylanase/acetyl xylan esterase, with a predicted molecular weight of 167,983 Da. The ORF contains several modules separated by glutamine-asparagine-rich linkers – two glycoside hydrolase 11 modules, a GH family 10 module, a CBM family 22 module, and a carbohydrate deacetylase at the C-terminal end. Additionally, a dockerin module is present indicating that it is cellulosome associated. This ORF was previously identified in the suppressive subtractive hybridization comparisons with *R. flavefaciens* JM1; [30]. Southern blots had indicated that both the GH 10 and 11 modules appeared in at least two separate EcoRI restriction fragments, and support the modular arrangement described in Table S2. A comparison of the modular organization inferred for xylanolytic enzymes from *R. flavefaciens* strains FD-1 and 17 is shown in Figure 3, which shows that while similar features are present, no two modular arrangements are identical between the two strains. The non-cellulosomal (ie. non dockerin-containing) enzyme XynA from *R. flavefaciens* 17 was previously reported to include a large NQ-rich linker, interconnecting GH11 and GH10 modules [21]. Although T-rich linkers are predominant in glycoside hydrolases from FD-1, three gene products were detected that carry NQ-rich linkers, or in one case a mixture of T-rich and NQ-rich linkers (Figure 4). The average amino acid composition of the five linkers within FD-1-ORF03896 (33% N, 35% Q, 10% W) was quite similar to that of the single large linker in *R. flavefaciens* 17 XynA (45% N, 26% Q, 16% W) [21]. The presence of the aromatic residue tryptophan in such linker regions is particularly unusual.

### Carbohydrate-binding modules

Permutations of glycoside hydrolases and carbohydrate-binding modules that occur in *R. flavefaciens* FD-1 are displayed in Table S4. The presence of CBMs in tandem with catalytic modules provides prolonged association with the substrate and can be found at either the N- or C-terminus of fiber-degrading enzymes. They are usually separated from the catalytic module by linker segments that are rich in proline, threonine and serine residues [42]. Over half of the identified CBMs in *R. flavefaciens* FD-1 are family 22 and 35 (Figure 1). Members of CBM families 3, 4, 6, 13, 32, and 48 were also identified. Additionally, there were 5 putative CBM modules that are presently unclassified in CAZy. The five CBM family 3 modules in the *R. flavefaciens* FD-1 genome were all found in tandem with a GH9 module. All five CBM3 modules fell within the CBM3c subfamily when compared to CBM3 modules from other organisms (Figure S2; Table S5). When paired with a particular subfamily of GH9, the CBM3c subfamily is thought to contribute in some cases to the property of processivity, allowing the enzyme to exhibit both endo- and exoglucanase activities [43], [44], [45]. The fact that none of the CBM3s map into subfamilies 3a or 3b indicates that none of them fulfill a defined binding capacity for crystalline cellulose. In ten ORFs, multiple CBMs are detected (ORF01222, ORF01406, ORF1541, ORF02983, ORF3116, ORF03219, ORF3447, ORF03865, ORF4012, and ORF04293). Of the 52 GHs found in tandem with CBMs, eight are of the GH43 family and all eight are encoded in tandem with dockerins. The majority of these encode arabinofuranosidases and arabinases. A close homologue was also found in ORF01571-ORF01570 for the new CBM family of cellulose-binding module that was identified adjacent to the GH44 catalytic module of *R. flavefaciens* 17 EndB (Cel44A) enzyme [23]. Another suspected new CBM is present in the EndA cellulase of *R. flavefaciens* 17 [22] and again a close homologue was detected in *R. flavefaciens* FD1 (ORF01388). Homologues (ORF03116) were also detected for the two new CBMs recently detected in the cell wall-attached, non-catalytic, dockerin-containing protein CttA that is encoded by the *sca* gene cluster [46].

### Phylogenetic relationships of GH5 and GH9 catalytic modules

The hypothetical translations representing the most prevalent glycoside hydrolases (GHs) detected (families 5 and 9) were aligned with other GH representatives from a variety of other fiber-degrading organisms using ClustalX [47]. The neighbor-joining tree produced from the GH family 5 alignment demonstrates an interesting phenomenon with relation to repeated modules within the same ORF (Figure S3; Table S6). Most known GH5 enzymes show cellulase activities, although numerous members of this family display xylanase and mannanase activities. In the GH family 5 phylogeny the two modules from ORF01388 appear less related to each other relative to the other representatives. The N-terminal module (ORF01388a) appears more closely related to the GH family 5 module detected in ORF00389 and ORF02868 and map together with known endoglucanases from *R. albus* and *R. flavefaciens* strain 17, whereas the C-terminal module from ORF01388 (ORF01388b) appears more closely related to the module detected in ORF00227, both of which are predicted mannanases. ORF03338 and ORF04165 map on a branch together with known xylanases.

As indicated in the previous section, five of the twelve GH family 9 modules, contained in ORF01045, ORF01053, ORF01132, ORF02970, and ORF02981, appear in tandem with CBM subfamily 3c modules. In these five processive endoglucanases, the family 3c CBMs appear adjacent to the GH family 9 module, towards the C-terminal end of the polypeptide (Table S2). The five GH9-CBM3c enzymes present one of the major thematic architectural schemes, which characterize this family of cellulases. The five GH9 catalytic modules map on one of the major branches of the phylogenetic tree (Figure S4; Table S7), together with two other GH9 modules (ORF01327 and ORF01899), each of which bears a module currently annotated as an unknown module in place of the CBM3c. It will be interesting in the future to determine whether this type of unknown module functions as a CBM and modulates the activity characteristics of the GH9 catalytic module. The remaining five family GH9 enzymes of *R. flavefaciens* FD-1 map on the phylogenetic tree on the second major branch together with GH9 enzymes of other bacterial species that include a family 4 CBM (Figure S4; Table S7). Indeed, all five of the latter enzymes bear an N-terminal CBM4, in accord with a second major thematic architectural scheme of the GH9 enzymes.

In contrast to the situation with polypeptides that carry GH10 and GH11 xylanase modules (Figure 3), there were rather few instances where GH5 or GH9 modules were combined with other catalytic modules in the same polypeptide. Thus for the six completed ORFs that include a GH5 module, and the four completed ORFs that include a GH9 module, these were the only identified catalytic module present, as opposed to some examples of multiple catalytic modules that occur in GH9 and GH5 enzymes of the *Clostridium thermocellum* cellulosome. Among incomplete ORFs, however, one (ORF01388) showed evidence of two GH5 modules of divergent specificities.

### Presence of cellulosome components in *R. flavefaciens* FD-1 – scaffoldins and complementary cohesin and dockerin modules

Scaffoldin sequences have been previously described and characterized for *R. flavefaciens* 17 [24], [25], [26], [48]. Using this sequence information, FastA searches of the *R. flavefaciens* FD-1 genome sequence were initially conducted in order to determine what components are maintained between *R. flavefaciens* strains, particularly components crucial to cellulosome formation. This led to the subsequent sequence and functional analyses between the scaffoldins of strains 17 and FD-1 described recently [28]. These studies showed a general similarity in cellulosome organization between the strains, including homologs of ScaA, ScaB, ScaC, and ScaE (see Table S8). However, the studies also revealed that ScaB from the FD-1 strain is comprised of two divergent cohesin types, unlike ScaB from strain 17, which is comprised of a single cohesin type. This description of scaffoldins in *R. flavefaciens* complements the previous identification of dockerin-like modules in both *R. flavefaciens* and *R. albus* [22], [49], [50], [51]. The presence of dockerin-containing proteins in *R. flavefaciens* FD-1 was expected, given the presence of cohesin-carrying scaffoldins. According to our analyses, the genome appears to encode for 225 dockerin-containing proteins (including those found in the aforementioned scaffoldins). The dockerins are found within almost all of the glycoside hydrolase-containing ORFs (Figure 1A and Table S2). Signal peptides were detected in all completed ORFs that include a dockerin, thus indicating secretion of these proteins (Table S8).

### Presence of non-carbohydrate active enzyme dockerin-containing ORFs

Analysis of the cellulosome associated ORFs revealed an astonishing number of non-carbohydrate acting enzymes linked to dockerins that made up 21% of the cellulosome associated ORFs. These ORFs include such modules as leucine rich repeats (LRR), transglutaminases, and serine protease inhibitors (SERPIN). Although these modules may not have a direct role in plant cell wall degradation, they could play a role in cell adhesion and protein-protein interactions. The LRR modules in particular have been shown to form protein-protein interactions [52], and thus they could act as a new type of cohesin.

### Comparing abundance of carbohydrate active enzymes among cellulolytic bacteria and the rumen metagenome

A recent study by Brulc et al. [53] sequenced the metagenome of the rumen of three steers, and looked specifically for carbohydrate active enzyme (CAZy) families in both the planktonic and fiber-adherent fractions of the rumen contents. The results of this study showed a large variety and abundance of GH families, most of which can also be found within the genomes of *R. flavefaciens* FD-1 and *C. thermocellum* (Table 2a). The most abundant GH families in both *R. flavefaciens* FD-1 and *C. thermocellum* are the GH families 5 and 9, whereas in the rumen metagenome the GH families 2 and 3 had the highest number of copies detected. The most likely reason for this is due to the fact that both *R. flavefaciens* and *C. thermocellum* specialize in crystalline cellulose degradation and thus two of the cellulase families are seen in the highest abundance, whereas in the rumen environment the population of cellulolytic bacteria is low compared to the overall microbial population and thus we see comparatively few cellulases detected. Alternatively, there may be difficulties in releasing of DNA from ruminococci as they are Gram positive and are in tight association with insoluble substrate. In the *C. thermocellum* and *R. flavefaciens* FD-1 genomes there are also many types of CBMs, though few were detected in the rumen metagenome (Table 3). The most abundant CBMs in the *R. flavefaciens* FD-1 genome were from family 22 (19 copies), and in the *C. thermocellum* genome the most abundant CBMs were from family 3 (23 copies). The total number of carbohydrate esterases (CE) detected in the rumen were comparable to the numbers seen in the *R. flavefaciens* and *C. thermocellum* genomes (Table 3). A single polysaccharide lyase (PL) was detected in the rumen samples, but the number of PLs compared to other carbohydrate active enzyme types was also rather low in both genomes (Table 3). The feature unique to *R. flavefaciens* FD-1, however, is the large copy number of dockerin sequences (225) compared to *C. thermocellum* (76 copies). Surprisingly, a mere 3 copies of dockerin modules were detected in the rumen metagenome (Table 3), which is most likely due to the rarity of cellulosome-based systems for plant cell wall degradation within the rumen community and the limits of the short pyrosequencing read lengths, as described by Brulc et al [53]. None of the dockerin modules from the rumen metagenome were consistent with those of *R. flavefaciens* FD-1.

**Table 2:** Comparison of copy numbers of glycoside hydrolase (GH) families in the genomes of *R. flavefaciens* FD-1 (*Rf*) and *Clostridium thermocellum* (*Ct*), and the pyrosequenced rumen metagenome.

CAZy Family	*Ct* genome	*Rf* FD-1 genome	Pooled Liquid	Fiber-Adherent 8	Fiber-Adherent 64	Fiber-Adherent 71
GH1	2	0	7	4	7	20
GH2	1	2	218	185	228	114
GH3	3	6	207	194	207	96
GH4	0	0	16	9	7	2
GH5	11	14	7	11	5	4
GH8	1	0	8	3	4	ND
GH9	16	12	7	6	6	5
GH10	6	6	10	5	7	4
GH11	1	11	2	ND	1	ND
GH13	2	4	47	36	37	39
GH15	1	0	ND	ND	ND	1
GH16	2	5	ND	ND	ND	1
GH18	3	1	2	ND	3	1
GH23	2	0	ND	ND	ND	ND
GH24	0	1	ND	ND	ND	ND
GH25	0	9	1	1	ND	ND
GH26	3	6	2	5	6	5
GH27	0	0	16	21	23	5
GH28	0	0	9	9	ND	ND
GH29	0	0	31	34	29	16
GH30	0	0	3	3	2	1
GH31	0	1	101	72	80	42
GH32	0	0	12	8	5	2
GH33	0	0	2	ND	1	1
GH35	0	0	21	8	9	10
GH36	0	1	47	43	48	48
GH38	0	0	22	16	19	11
GH39	0	0	2	3	3	1
GH42	0	1	10	7	15	13
GH43	6	10	68	72	69	35
GH44	1	2	ND	ND	ND	ND
GH48	2	1	ND	ND	1	ND
GH51	1	0	73	54	86	44
GH53	1	1	15	16	18	17
GH54	0	0	ND	ND	3	1
GH57	0	0	2	ND	ND	1
GH74	1	1	ND	ND	ND	ND
GH77	0	1	ND	ND	2	ND
GH78	0	0	41	37	38	18
GH81	1	0	ND	ND	ND	ND
GH92	0	0	43	67	66	28
GH94	3	1	ND	ND	ND	ND
GH95	0	1	ND	ND	ND	ND
GH97	0	2	47	67	59	20
GH105	0	1	ND	ND	ND	ND
GH106	0	0	9	9	11	4
Total GH	70	101	1108	1005	1105	610

**Table 3:** Comparison of copy numbers of carbohydrate active enzyme families in the genomes of *R. flavefaciens* FD-1 (*Rf*) and *Clostridium thermocellum* (*Ct*), and the pyrosequenced rumen metagenome.

CAZy Family	*Ct* genome	*Rf* FD-1 genome	Pooled Liquid	Fiber-Adherent 8	Fiber-Adherent 64	Fiber-Adherent 71
CBM3	23	5	ND	ND	ND	ND
CBM4	4	7	ND	ND	ND	ND
CBM6	10	3	ND	1	ND	ND
CBM9	1	0	ND	ND	ND	ND
CBM11	1	0	ND	ND	ND	ND
CBM13	2	10	1	ND	1	2
CBM22	4	19	ND	ND	ND	ND
CBM25	2	0	ND	ND	ND	ND
CBM30	1	0	ND	ND	ND	ND
CBM32	1	3	ND	3	ND	1
CBM35	7	13	ND	ND	ND	ND
CBM42	4	0	ND	ND	ND	ND
CBM44	1	0	ND	ND	ND	ND
CBM48	1	2	ND	ND	ND	ND
CBM_NC*	0	6	ND	ND	ND	ND
CE1	3	8	5	10	22	8
CE2	1	3	1	1	1	ND
CE3	1	3	ND	ND	ND	ND
CE4	3	5	6	2	5	4
CE6	0	0	ND	ND	ND	1
CE7	1	0	ND	2	3	1
CE8	1	1	ND	ND	ND	ND
CE9	2	0	ND	ND	ND	ND
CE12	1	5	ND	ND	ND	ND
CE15	0	1	ND	ND	ND	ND
PL1	2	6	ND	ND	ND	ND
PL9	0	1	ND	1	ND	ND
PL11	1	6	ND	ND	ND	ND
COH	29	18	ND	ND	ND	ND
DOC	76	225	2	ND	1	ND
Total CBM	62	68	1	4	1	3
Total CE	13	26	12	15	31	14
Total PL	3	13	0	1	0	0

* Not characterized.

### Microarray gene expression profiling upon growth of *R. flavefaciens* FD-1 on cellulose or cellobiose

A clone-based cDNA microarray was created by amplifying clone inserts from the most recent library used in the sequencing of the *R. flavefaciens* FD-1 genome to compare gene expression when *R. flavefaciens* FD-1 was grown on cellulose or cellobiose as a carbon and energy substrate. Clone sequences encoding ORFs believed to be associated with the cellulosome or involved in degradation of polysaccharides, were identified by BLAST searches of a local database and by the genome annotation of *R. flavefaciens* FD-1, which was provided by TIGR's Manatee annotation engine. Normalized signal ratios for each spot corresponding to ORFs involved in polysaccharide degradation were calculated representing gene expression for cells grown on cellulose compared to those grown on cellobiose. Clones with an FDR-adjusted p-value less than 0.5 were considered significant. A transcript was considered to be up-regulated if the average of the signal ratio for the ORF was 2-fold or greater, and considered down-regulated if the average of the signal ratio was 0.5-fold or less. The expression of any gene transcript falling below 2-fold and above 0.5-fold was considered to be unaffected by the substrate [54].

Cellulosome-associated ORFs included any ORF that encoded a dockerin module. As reported above, the draft genome of *R. flavefaciens* FD-1 encodes 225 predicted dockerin modules. These ORFs, the number of clones in each ORF that was included on the microarray, and the corresponding average signal ratios can be seen in Table S9. Of these 225 cellulosome-associated ORFs: 56 were up-regulated, 14 were down-regulated, 135 were unaffected, and 20 were not represented on the microarray. The 20 dockerin-containing ORFs not represented on the microarray due to the inclusion of only 2× coverage of the genome on the microarrays included numerous additional modules and/or domains: 15 ORFs contain domains of unknown function, the remaining five ORFs contain a serpin, a leucine-rich (LRR) domain, a CBM4-GH9, a CBM35-GH26, and a GH18.

The Sca cluster in *R. flavefaciens* FD-1, which includes the main scaffoldins: ScaA, ScaB and ScaC, was significantly up-regulated *en bloc* approximately 4.5 fold, which suggests that these genes are co-expressed either as a polycistronic mRNA or sharing the same regulator with similar affinity for these genes. The last two genes of the Sca cluster, *cttA* and ScaE, do not appear to be co-expressed with ScaA, ScaB, and ScaC, and appear to have different regulators. The last scaffoldin gene of the cluster – the putative cellulosome anchoring scaffoldin, ScaE, had significant relative expression of 2.94. The product of the linked gene *cttA*, exhibited a relative expression of 0.75 fold and thus appeared to be unaffected by the substrate. Fold changes for ScaA, ScaB, ScaC, and ScaE can be seen in Table S9. Of the other putative scaffoldins, ORF00794, ORF04069, and ORF04333 were unaffected, ORF03129 appears to be down-regulated (0.47 fold) and ORF01453 was significantly up-regulated 4.27 fold.

Results for some other genes were of particular interest. ORF01132 contains a family-9 processive endoglucanase, which has been described as an important cellulosome component of other species of bacteria [55], [56], [57]. This processive endoglucanase was up-regulated 4.49 fold. CelA (ORF00507) and CelD (ORF01899) were unaffected (1.10 fold) and up-regulated (4.93 fold) respectively (Table S10), which is consistent with previous results [18], [58]. CelB (ORF01869) was unaffected (1.13 fold; Table S9), which contradicts previous data that indicated that it was inducible by cellulose [17], [58], [59]. A putative exo-acting GH48 of the *R. flavefaciens* FD-1 genome (ORF03925) was unaffected by the substrate, unlike the observed up-regulation of the *C. thermocellum* cellulosomal GH48 [60], [61], [62]. These apparently different expression patterns are likely due to the different environmental conditions to which these two bacteria are exposed, including oxygen concentrations and plant cell wall substrate type.

**Figure 5:**
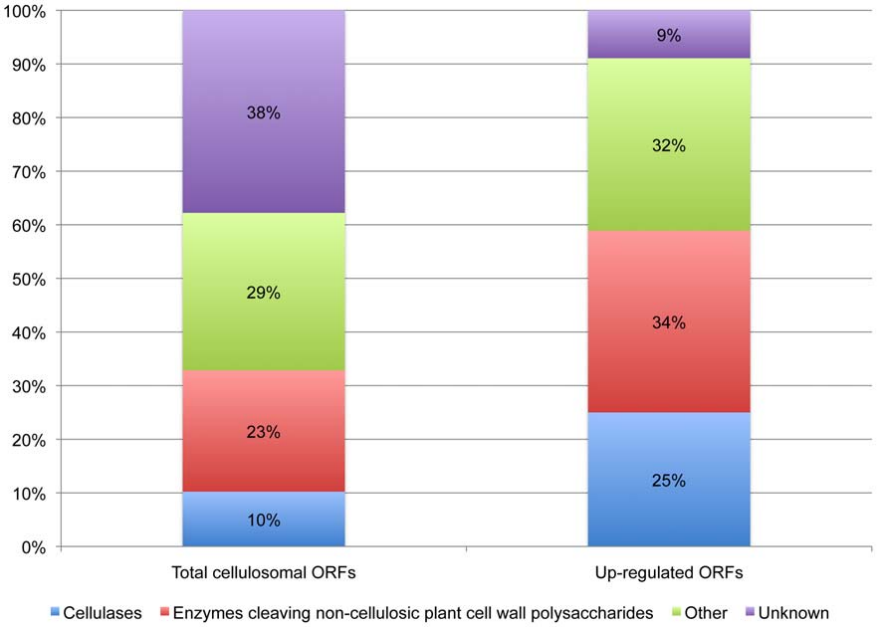
Proportions of cellulases, enzymes cleaving non-cellulosic plant cell wall polysaccharides (including carbohydrate esterases) and other predicted ORFs among the total cellulosome-associated genes and the up-regulated cellulosome-associated ORFs. Up-regulated genes are those dockerin-containing ORFs that have fold changes of 2-fold or greater when grown on cellulose. For the purposes of this work, the putative cellulases include any ORF containing glycoside hydrolase (GH) families 5, 8, 9, and 48. The enzymes cleaving non-cellulosic plant cell wall polysaccharides (mainly hemicellulases) include ORFs containing GH families 10, 11, 16, 26, 43, 44, 53, 74 105, some subfamilies of GH5, all families of polysaccharide lysases (PL) and carbohydrate esterases (CE). ORFs that did not have any significant hits in the database are grouped as “unknown,” and ORFs that do not fall into any of the previous categories are grouped as “other.” Putative β-glucosidases and β-xylosidases were ORFs containing sequences consistent with GH family 3.

The proportion of cellulases compared to enzymes cleaving non-cellulosic plant cell wall polysaccharides and other ORFs within the cellulosome-associated ORFs that encode a dockerin module is shown in Figure 5. Cellulases (GH families 5, 8, 9, and 48) made up 25% of the up-regulated ORFs compared to 10% of all dockerin-encoding ORFs. The enzymes cleaving non-cellulosic plant cell wall polysaccharides made up 23% of all dockerin-encoding ORFs and 34% of the up-regulated cellulosomal ORFs. Enzymes cleaving non-cellulosic plant cell wall polysaccharides also accounted for some of the highest relative expression when grown on cellulose. The three cellulosome-associated ORFs with the highest regulation were the multi-modular xylanases: SIGN-GH11-CBM22-GH10-DOC-CBM22-CE4 (ORF01222), SIGN-GH11-CBM22-GH10-DOC1-GH11-CE4 (ORF03896) and SIGN-GH11-CBM22-DOC-GH11-CE3 (ORF01315) with respective significant relative expression levels of approximately 63, 50, and 25 fold above those of cellobiose-grown cells. The predicted ORF03896 product is one of the ORFs containing NQ-rich, rather than T-rich linker sequences. Such linkers have been reported previously in only one non-cellulosomal xylanase from *R. flavefaciens* 17 that also included GH11 and GH10 catalytic modules [21].

Non-cellulosomal open reading frames, i.e. those ORFs that do not contain a dockerin module, are listed in Table S10. Of the 71 genes included in this list, 4 (6%) were up-regulated, 6 (8%) were down-regulated, 54 (76%) were unaffected, and 7 (10%) were not included on the microarray. The genes that are not on the microarray are composed of five GH family 25 modules (two of which are found in a single ORF), a GH family 3 module, a CBM family 22 module, and a glycosyltransferase family 28 module.

### Comparison of relative gene expression using quantitative real-time reverse transcriptase PCR

RNA samples that were extracted from cellulose- and cellobiose-grown cultures of *R. flavefaciens* FD-1 were used for quantitative real-time reverse transcriptase PCR (qRT-PCR), in order to validate the microarray data. The same RNA samples that were used for the microarray experiments were used for these qRT-PCR experiments. Five genes of particular interest to us were selected based on their putative function and/or dramatic change in relative gene expression between the two conditions. These genes include: a multi-modular xylanase (ORF03896), a GH family 9 processive endoglucanase (ORF01132), a GH family 48 exoglucanase (ORF03925), ScaA (ORF03114), and a highly down-regulated dockerin-containing gene of unknown function (ORF04112). The primer sequences for these genes and the normalization gene, *gyrA*, are listed in Table S11. The gene, *gyrA*, was chosen as a reference gene to normalize the qRT-PCR data because it did not have a statistically significant change in expression, based on the results of the microarray experiments, and it has been commonly used as a normalization gene for bacteria in other studies [63], [64], [65], [66]. The 16S gene was also intended for use as a normalization gene, but was found to produce inconsistent results with these samples (data not shown). A relative standard curve method was used to determine the relative expression of these genes (Applied Biosystems User Bulletin 2; [67]). Serial dilutions of *R. flavefaciens* FD-1 genomic DNA were used to generate standard curves to determine the relative copy numbers of the cDNA samples by correlating the samples to particular concentration.

**Figure 6:**
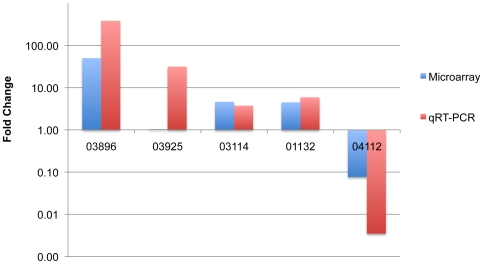
Comparison of microarray data to qRT-PCR data in terms of relative expression (fold change) of five selected ORFs. Each number on the x-axis corresponds to the ORF designation assigned by TIGR's annotation engine.

The qPCR data confirmed the up-regulation of three ORFs, and the down-regulation of one, although the magnitude of the regulatory changes was greater than in the microarray study (Table S12, Figure 6). In the case of the GH48 enzyme encoded by ORF03925, up-regulation was detected by qPCR but not by microarray. The difference between the qPCR and microarray data for ORF03925 could be due to decreased sensitivity of the microarray or could be explained by a low correlation between microarray and qPCR results in genes that exhibit low changes in expression between treatments [68]. The qPCR results, which indicate up-regulation of the GH48 enzyme, are more in accord with the previously reported data for the orthologous *C. thermocellum* enzyme [60], [61], [62].

### Conclusion

Portions of the cellulolytic enzyme system from *R. flavefaciens* strain FD-1 have been previously characterized as a variety of exo-β-1,4-glucanases, endo-β-1,4-glucanases, and cellodextrinases [9], [10], [11], [12]. Evidence was found for two major endo-β-1,4-glucanase complexes, one including at least 13, and the other at least 5, electrophoretically separable endo-β-1,4-glucanase activities [12]. This is consistent with the large diversity of genes found here that have the potential to encode endoglucanase activity.

Complex multi-modular organization, involving multiple catalytic and substrate-binding modules within the same polypeptide, has been documented previously for plant cell wall degrading enzymes, especially xylanases, from the related strain *R. flavefaciens* 17 [19], [20], [21], [22]. This genomic analysis establishes that such organization is a common feature in particular of xylanases and esterases from *R. flavefaciens* FD-1. Interestingly, however, despite many close similarities and common features, it was not always possible to identify precise homologues of these multi-modular enzymes between the two strains. Of the five xylanases and esterases characterized from *R. flavefaciens* 17, for example, none showed an exact match in modular structure to a homologue in strain FD-1. *R. flavefaciens* FD-1 ORF02390, for example, shares close homology with *R. flavefaciens* 17 CesA (CE3B) through its family 3 esterase and an unknown domain, at the N and C terminus respectively, but includes an additional CBM22 module. *R. flavefaciens* FD-1 ORF03896 and *R. flavefaciens* 17 XynA are superficially similar in carrying GH11 and GH10 xylanase modules and NQ-rich linkers, but the FD-1 ‘superzyme’ differs in carrying additional CE4 and GH11 modules and a dockerin. This suggests that there is considerable evolutionary plasticity in the modular structures of these enzymes, with domain shuffling occuring readily to produce new variations within a given strain [69]. Close homologues were, however, observed for certain enzymes, such as *R. flavefaciens* 17 EndB (Cel44A).

Close similarities in gene order between *R. flavefaciens* FD-1 and 17 were identified for two important chromosomal regions concerned with the utilization of plant cell wall polysaccharides. Conservation of the four key cellulosomal scaffoldin genes within the *sca* cluster, *scaC*, *scaA*, *scaB*, and *scaE* was reported recently [28]. An additional gene *cttA*, found within the cluster whose product is concerned with cell adhesion to cellulose [46] was also conserved. The microarray results also showed that when grown on cellulose, *scaA*, *scaB*, and *scaC* in *R. flavefaciens* FD-1 all have similar signal ratios (approximately 4.5 fold above that of cellobiose) implying that they are transcribed together, forming an operon. Compared to *R. flavefaciens* 17, however, differences were observed at the level of modular organization with the *R. flavefaciens* FD-1 ScaA protein carrying one fewer cohesin module than ScaA from *R. flavefaciens* 17, and with the FD-1 ScaB protein exhibiting two types of cohesin [28]. Along with the frequent differences in enzyme modular structures noted above, this suggests that there may be many differences in the detailed organization of the cellulolytic enzyme complexes between the two strains. We were also able to demonstrate a region of synteny between genes concerned with the utilization of xylo-oligosaccharides [40] that include the β-xylosidase, xylose isomerase and components of an ABC transporter system. In both of the strains, this region was found to be flanked by genes that encode cellulosomal enzymes associated with the degradation of hemicellulose.

The variety of dockerin-containing enzymes in the *R. flavefaciens* FD-1 genome suggests that there are many configurations that the cellulosome can assume. Expression profiling using microarrays, and verified by qRT-PCR, revealed that the type of substrate utilized by *R. flavefaciens* FD-1 drives the potential cellulosome composition. This is expected to result in the production of an incredibly heterogeneous collection of cellulosomes during the course of plant cell wall polysaccharide degradation. It is interesting to note that the minority (33%) of the 225 dockerin containing ORFs was made up of the cellulases and enzymes active against non-cellulosic structural polysaccharides (Figure 5). However, when looking exclusively at the up-regulated dockerin-containing ORFs, the cellulases and enzymes active against non-cellulosic structural polysaccharides made up 59% of the ORFs. This indicates that when grown on a cellulose substrate, *R. flavefaciens* FD-1 preferentially expresses enzymes that are designed for hydrolysis of complex carbohydrates. Curiously, of these ORFs, the most highly up-regulated enzymes during growth on cellulose were the hemicellulases, not the cellulases. The three most highly up-regulated enzymes show remarkably complex structures, each with three catalytic modules and one or more CBMs. Interestingly, previous studies on *R. flavefaciens* 17 showed by zymogram analysis that high molecular weight xylanase polypeptides (>70 kDa) were expressed during growth on cellulose, or in some cases only on xylan or oat straw, but not on cellobiose [70]. A likely explanation for these findings is that, in nature, *R. flavefaciens* rarely comes across pure cellulose, because cellulose is typically accompanied by other plant cell wall polysaccharides. Therefore, in order to depolymerize these other non-cellulosic components and gain access to the cellulose, the microbe would need to use enzymes other than the cellulases to remove the non-cellulosic plant cell wall components. In addition, many *R. flavefaciens* strains are able to utilize products from xylan, as well as cellulose breakdown, for growth [40].

## Materials and Methods

### Organisms and culture conditions

*R. flavefaciens* FD-1 from the Department of Animal Sciences culture collection was used as the source of genomic DNA in library construction and was cultivated in a defined medium as described by Antonopoulos et al [71]. Cells were grown at 37°C in crimped butyl rubber stoppered bottles (Bellco Glass, Inc., Vineland, NJ) saturated with 95% CO_2_/5% H_2_ atmosphere. Stock cultures were maintained on solid agar slants at −120°C. *Escherichia coli* One Shot^®^ MAX Efficiency^®^ DH10B™ (Invitrogen, Carlsbad, CA) was used as the host in library constructions. Transformed *E. coli* cells were grown in LB medium supplemented with 100 µg/mL of ampicillin (Sigma-Aldrich, St. Louis, MO) for selection and maintenance of plasmids.

### Genomic DNA extraction and shotgun library construction

Extraction of genomic DNA from *R. flavefaciens* FD-1 has been described previously [29]. Chromosomal DNA extracted from *R. flavefaciens* FD-1 was subjected to high-pressure shearing (N_2_) via a nebulizer and then treated with Bal31 nuclease (New England Biolabs, Beverly, MA) to remove single-strand overhangs. This pool of sheared DNA fragments was then size fractionated (i.e. fragments between 1.5–3 kb were gel excised), gel purified, and subjected to a series of “polishing” reactions by T4 DNA polymerase and Klenow fragment (Invitrogen, Carlsbad, CA). Shrimp alkaline phosphatase was then used in a dephosphorylation treatment to remove 5′-phosphoryl groups (Roche Applied Science, Penzberg, Germany). Cloning of the sheared, “polished”, and dephosphorylated fragments was performed using the pCR^®^4Blunt-TOPO^®^ vector (Invitrogen, Carlsbad, CA). Transformation of *Escherichia coli* One Shot^®^ MAX Efficiency^®^ DH10B™ cells (Invitrogen, Carlsbad, CA) was conducted by electroporation (transformation efficiency of 10^7^ transformants/µg DNA) followed by immediate plating onto ampicillin-supplemented LB agar plates.

### Sequencing and assembly of contigs

In total 11,520 transformants were picked robotically using a QPix robot (Genetix, UK) and transferred into starter freeze-down media in 384-well plates. Following overnight incubation the plates were transferred to a −80°C freezer for storage. To sequence the selected clones, they were transferred from the frozen stocks to 96-well plates, grown overnight, and a QIAGEN 9600 robotic system was then used to extract the plasmids. Big Dye terminator chemistry, in conjunction with standard M13-based forward and reverse primers based on the pCR^®^4Blunt-TOPO^®^ vector, was used for the sequencing reactions on an ABI 3700 capillary system (conducted at the W. M. Keck Center for Comparative and Functional Genomics on the UIUC campus). In addition to Sanger sequencing, extracted genomic DNA was subjected to a pyrosequencing run on a Roche 454 GX-FLX system at the W. M. Keck Center for Comparative and Functional Genomics on the UIUC campus. Vector trimming, sequence editing, and quality control was handled by the Bioinformatics Unit of the W.M. Keck Center, as well as the maintenance of the sequence database on their servers. Base calling and contig assembly were conducted using Phred/Phrap and visualized with Consed [31], [32], [33]. Subsequent, manual linking of contigs was performed using Consed [33]. The genome sequence (119 contigs) has been deposited into the DDBJ/EMBL/GenBank databases under the accession number ACOK00000000.

### ORF identification and annotation

Following contig assembly and vector trimming, the contigs comprising the assembly were used in BlastX comparisons with the locally stored non-redundant GenBank database (a cut-off E-value of e^−05^ was used for the initial survey; [72]). These preliminary sequence identifications were supplemented by focused searches of the assembly using the FastA collection of programs and individual sequences of interest as queries [73]. Identification and annotation of putative genes from the *R. flavefaciens* FD-1 sequence assembly was also performed by TIGR's Annotation Engine (a service funded by the US Department of Energy; see TIGR's Annotation Engine website for further details at http://www.tigr.org/edutrain/training/annotation_engine.shtml). The Glimmer software package was used initially to identify likely candidates for genes [74], [75]. Several searches were then performed using the candidate ORFs identified by Glimmer as queries. BLAST-Extend-Repraze (BER) was used to search TIGR's non-redundant amino acid database (nraa) containing all proteins available from GenBank, PIR, SWISS-PROT, and TIGR's Comprehensive Microbial Resource (CMR) database [72]. A second round of searches were performed against hidden Markov models using the hmmpfam program [76]. AutoAnnotate was then used to analyze the BER and HMM searches and to assign a function to each of the sequences.

### Organisms and growth conditions for microarrays

*Ruminococcus flavefaciens* FD-1 [4] was grown anaerobically in defined media containing either 0.1% w/v pebble milled cellulose (filter paper) or 0.4% w/v cellobiose, 0.2% w/v Bacto-Tryptone, 0.1% w/v Bacto-Yeast Extract, 5% v/v mineral solution 1 and 2 [77], 1% v/v volatile fatty acid (VFA) solution [78], 0.0001% w/v resazurin, 0.4% w/v NaHCO_3_ and 0.025% w/v cysteine-sulfide. Cultures were grown at 37°C in butyl rubber-stoppered flasks under a 95% CO_2_/5% H_2_ atmosphere. Growth curves were determined for both cellobiose and cellulose cultures by measuring optical density at 600 nm for cellobiose and by monitoring substrate disappearance for cellulose (data not shown). Growth curve data were also compared to growth curves performed by Odenyo et al. (1992; 1994). Media containing cellobiose was grown for approximately 9 h late to log phase [79]. Media containing cellulose was grown for approximately 19 h to late log phase (Odenyo, 1992 PhD thesis, University of Illinois at Urbana-Champaign). Four independent replicate cultures were grown in triplicate for each substrate.

### RNA extractions

Cells were pelleted for RNA extraction by first adding 75 ml of ice-cold RNase free DEPC-treated water per 50 ml of cell culture, placing on ice for 5 min, then centrifuging at 4°C for 5 min at 2,800 x g. Supernatant fluids were removed, and cells were resuspended in 2.5 ml ice-cold RNase free DEPC-treated water. One ml aliquots of the cell suspension were transferred to 2.0 ml screw cap tubes and centrifuged at room temperature for 15 s at 13,000 x g. Supernatant fluids were removed, and cell pellets were stored at −20°C until needed for RNA extraction. RNA was extracted from the cell pellets using the RNeasy Kit – Yeast III Protocol (Qiagen, Valencia, CA) according to the manufacturer's instructions. A mini-bead beater set to homogenize was used as part of the lysis process. Cells were homogenized in the mini-bead beater 3 times for 2 min each and cooled on ice for 2 min between each homogenization. A DNase digestion was carried out using the On-column DNase Digestion with RNase-free DNase Set (Qiagen, Valencia, CA) according to the manufacturer's instructions. RNA quality was assessed by 1% agarose gel electrophoresis after treatment with an equivalent volume of 10 M urea and heating at 70°C for 5 min to eliminate any secondary structure. RNA concentrations were estimated by absorbance at 260 nm using a Beckman DU-7000 spectrophotometer.

### Microarray design and construction

6,144 PCR-amplified clone inserts from the RF03 library were spotted in duplicate onto slides at the W.M Keck Center for Comparative and Functional Genomics, using a Gene Machines OmniGrid 100 Microarrayer (Genomic Solutions, Ann Arbor, MI). Controls consisted of *R. flavefaciens* FD-1 genomic DNA, *R. flavefaciens* FD-1 16S V3 rDNA tag, *E. coli* genomic DNA, *E. coli* 16S V3 rDNA tag, and a no template control consisting of the buffer only. The RF03 library is the most recent clone library to be included into the *R. flavefaciens* FD-1 draft genome, and therefore the microarrays contain the *R. flavefaciens* FD-1 genome at approximately 2× coverage (Antonopoulos 2004 PhD dissertation, University of Illinois at Urbana-Champaign).

### Aminoallyl-labeling of RNA

RNA was labeled by reverse transcription as follows: 5 µg of RNA was mixed with 2 µl of random hexamer primers (3 mg/ml) (Invitrogen, Carlsbad, CA) in a final volume of 18.5 µl, and incubated at 70°C for 10 min then placed on ice. The labeling reaction (0.5 µl of RNase inhibitor (Invitrogen, Carlsbad, CA), 6 µl of 5× First Strand Buffer (Invitrogen, Carlsbad, CA), 3 µl of 0.1 M DTT (Invitrogen, Carlsbad, CA), 0.6 µl 50× aminoallyl-dNTP mix [25 mM dATP, 25 mM dCTP, 25 mM dGTP, 5 mM dTTP, 7 mM aa-dUTP], and 2 µl SuperScript III RT (200 U/∝L) [Invitrogen, Carlsbad, CA]) was added to the mixture and the reaction was incubated at 46°C overnight. RNA was hydrolyzed by addition of 10 µl each of 1 M NaOH and 0.5 M EDTA, followed by incubation at 65°C for 15 min. The reaction was neutralized by addition of 10 µl of 1 M HCl. Ten µl of 3 M Na•Acetate, pH 5.2 was added to facilitate binding of cDNA to the Qiagen column. Unincorporated aa-dUTP and free amines were removed using a Qiagen QIAquick PCR Purification Kit protocol, according to manufacturer's instructions, and substituting phosphate wash buffer [5 mM KPO4 pH 8.5, 80% EtOH] for Buffer PE and phosphate elution buffer [4 mM KPO_4_, pH 8.5] for Buffer EB. The elution step was carried out twice with 30 µl of phosphate elution buffer.

### Dye incorporation

Purified cDNA samples were dried in an Eppendorf Vacufuge for approximately 1 h. Alexa-fluor 555 and 647 (Invitrogen, Carlsbad, CA), which correspond to Cy3 and Cy5 respectively, were resuspended in 9 µl of 0.1 M Na_2_CO_3_ then transferred to the cDNA sample and mixed. The reactions were incubated in the dark for 1 h at room temperature. Uncoupled dyes were removed using the Qiagen PCR Purification Kit according to the manufacturer's instructions. The elution step was carried out twice with 40 µl of Buffer EB. The labeling reactions were analyzed by measuring absorbance at 260 nm and either 550 nm (Alexa-555) or 650 nm (Alexa-647). The amount in pmol of cDNA, incorporated Alexa-555 and Alexa-647 dyes were calculated, and a cDNA/dye ratio was determined for each sample; greater than 30 pmol of dye incorporation and a ratio less than 50 nucleotides/dye molecule is optimal. Samples were dried to completion in an Eppendorf Vacufuge.

### Microarray hybridization, washing and scanning

The microarrays were prepared for hybridization by first binding the DNA to the slides by rehydrating the microarray over steam, drying on a heat block (∼70°C), then placing in a UV stratalinker at 300 mJ. Microarrays were washed vigourously in 0.2% SDS (w/v), then twice in ddH_2_O for two minutes each. The microarrays were added to preheated (42°C) pre-hybridization buffer [20% Formamide (v/v), 5× Denhardt's, 6× SSC, 0.1% SDS (w/v), 25 µg/ml tRNA] and incubated at 42°C for 45 min, shaking occasionally. Microarrays were washed in ddH_2_O five times, once in isopropanol, then dried immediately by centrifugation.

Hybridization was performed with four biological replicates, which included a dye swap. Labeled cDNA was resuspended in 80 µl of preheated (68°C for 15 minutes) SlideHyb #1 (Ambion). Samples were heated at 95°C for 5 min, then all 80 µl was applied to the microarray slide. 10 µl of ddH_2_O was added to the hydration chambers of the waterproof Corning hybridization chamber (Corning Life Science) to ensure a humid environment. Microarrays were allowed to hybridize in a 42°C water bath in the dark for approximately 3 d.

Coverslips were removed after hybridization and the microarrays were washed in 1× SSC, 0.2% w/v SDS at 42°C, followed by 0.1× SSC, 0.2% w/v SDS at room temperature, and twice in 0.1× SSC at room temperature agitating for 5 min at each step. The microarrays were dried immediately by centrifugation and scanned using an Axon GenePix 4000B scanner (Molecular Devices).

### Microarray analysis

Slide images were analyzed using the spot finding feature of GenePix Pro 6.0 (Molecular Devices). Microarrays were manually edited and aberrant spots were flagged for exclusion later on in the analysis. The resulting files were loaded into GeneSpring GX 7.3 (Agilent Technologies). The microarrays were normalized using Lowess normalization and the t-test p-values were FDR adjusted such that spots with an FDR p-value of less than 0.05 were considered significant. Each spot, which corresponds to a sequence from the genome assembly, was mapped back to the most current *R. flavefaciens* FD-1 genome assembly. Fold changes of dockerin-containing ORFs and glycoside hydrolase-containing ORFs were analyzed using Microsoft Excel to calculate the average of the signal ratios. Fold changes greater than or equal to 2-fold were considered up-regulated and fold changes less than or equal to 0.5-fold were considered down-regulated [54]. Microarray data were submitted to the Gene Expression Omnibus (GEO) in accordance with MIAME standards under GEO accession number GSE15916.

### Quantitative real time RT-PCR

qRT-PCR was performed to confirm the gene expression results of the microarrays. Aliquots of 0.5 µg of RNA were converted to cDNA via the SuperScript III First Strand Synthesis SuperMix for qRT-PCR (Invitrogen, Carlsbad, CA) according to the manufacturer's instuctions. Each qPCR reaction consisted of 1× SYBR Green Master Mix (Applied Biosystems, Foster City, CA), 50 nM of forward primer, and 50 nM of reverse primer to which 1 µl of undiluted cDNA was added. All reactions were done in triplicate. Primers were designed for ORFs 1132, 3114, 3896, 3925, and 4112, as well as for *gyrA* (ORF02752), using Primer3 (http://workbench.sdsc.edu/) and synthesized by Sigma-Genosys (Table S11). The reactions were run on an ABI 7900HT Sequence Detection System (Applied Biosystems, Foster City, CA). The cycling conditions consisted of a hold at 50°C for 2 min, a hold at 95°C for 10 min, 40 cycles of 95°C for 15 s and 60°C for 1 min, and then a dissociation profile of 95°C for 15 s, 60°C for 15 s, and 95°C for 15 s. The relative standard curve method was used to determine the relative amount of gene expression in *R. flavefaciens* FD-1 when grown on cellulose or cellobiose. *R. flavefaciens* FD-1 genomic DNA was serially diluted in TE Buffer, pH 8.0 from 10^−1^ to 10^−6^ to be used as standards for the standard curves from which the quantities of cDNA in the samples were determined. The *gyrA* gene (ORF02752) was used to normalize the Ct values from each sample prior to comparison [64], [80].

## Supporting Information

Figure S1 Unrooted dendrogram of the putative glycoside hydrolase family 48 modules (pfam02011) detected in R. flavefaciens FD-1 compared with those of other organisms.
(0.70 MB TIF)

Figure S2 Unrooted dendrogram of putative family 3 carbohydrate-binding modules detected in R. flavefaciens FD-1 compared with those from other organisms. “RfFD-1” refers to R. flavefaciens FD-1, and is followed by ORF designation number assigned by TIGR's Annotation Engine. “Clotm” refers to C. thermocellum, “Rumal” refers to R. albus, and these are followed by the enzyme name.
(0.80 MB TIF)

Figure S3 Unrooted dendrogram of glycoside hydrolase family 5 modules detected in R. flavefaciens FD-1 compared with those from other organisms. “Rf” refers to R. flavefaciens, and the ORF number refers to TIGR's Annotation Engine designation. The scale bar indicates the percentage (0.1) of amino acid substitutions.
(0.82 MB TIF)

Figure S4 Unrooted dendrogram of glycoside hydrolase family 9 modules detected in R. flavefaciens FD-1 compared with those from other organisms. “Rf” refers to R. flavefaciens, and the ORF number refers to TIGR's Annotation Engine designation. The scale bar indicates the percentage (0.1) of amino acid substitutions.
(0.77 MB TIF)

Table S1 (0.04 MB XLS)

Table S2 (0.04 MB XLS)

Table S3 (0.09 MB DOC)

Table S4 (0.02 MB XLS)

Table S5 (0.11 MB DOC)

Table S6 (0.14 MB DOC)

Table S7 (0.10 MB DOC)

Table S8 (0.03 MB XLS)

Table S9 (0.05 MB XLS)

Table S10 (0.02 MB XLS)

Table S11 (0.02 MB XLS)

Table S12 (0.02 MB XLS)
