# Supplementary figures and images for: Diversity and Strain Specificity of Plant Cell Wall Degrading Enzymes Revealed by the Draft Genome of Ruminococcus flavefaciens FD-1
Source: PLoS One. 2009 Aug 14;4(8):e6650. doi: 10.1371/journal.pone.0006650 (PMC2721979; doi:10.1371/journal.pone.0006650)

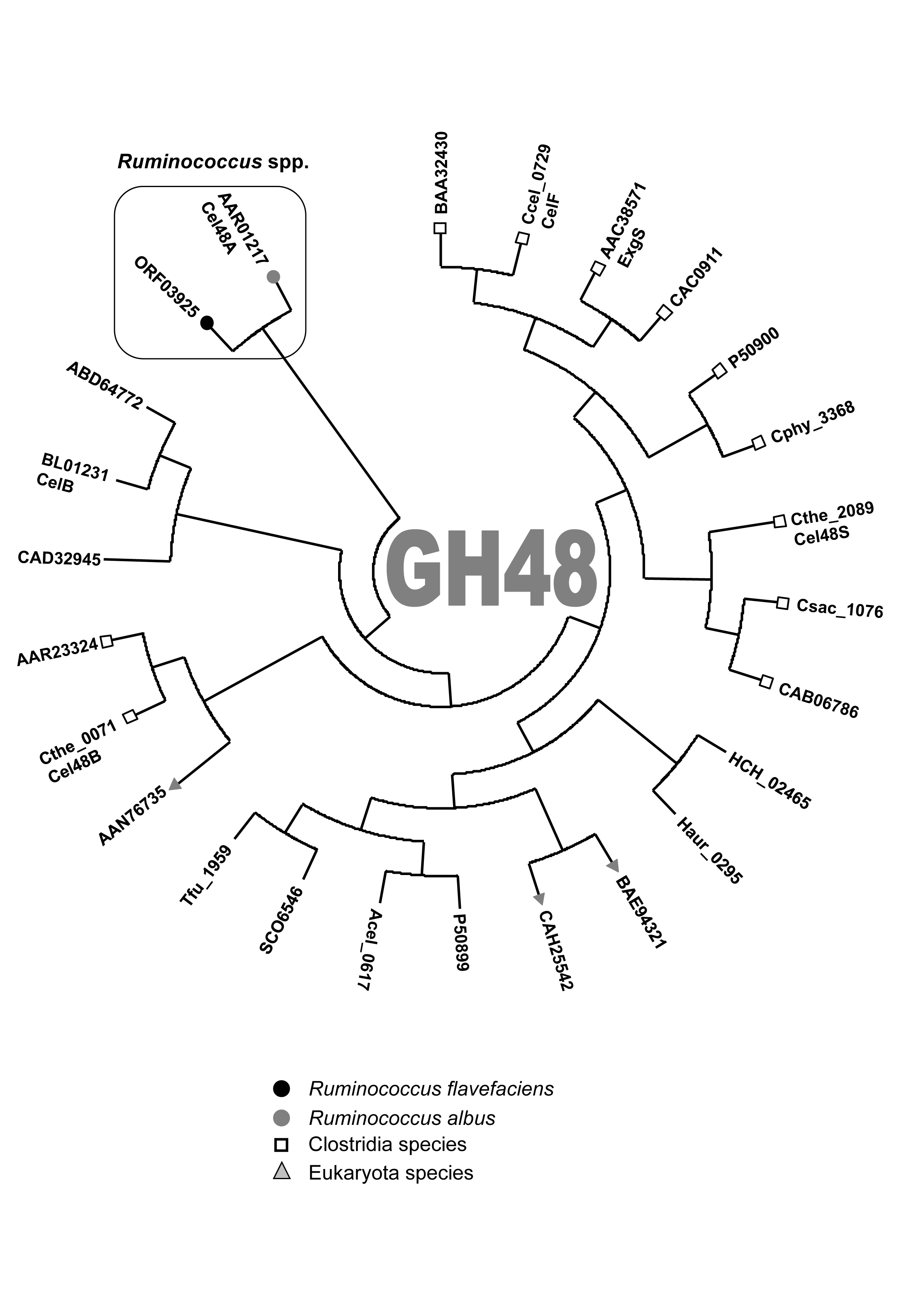

Supplement: Figure S1 — Unrooted dendrogram of the putative glycoside hydrolase family 48 modules (pfam02011) detected in R. flavefaciens FD-1 compared with those of other organisms. (0.70 MB TIF) [file pone.0006650.s001.tif]

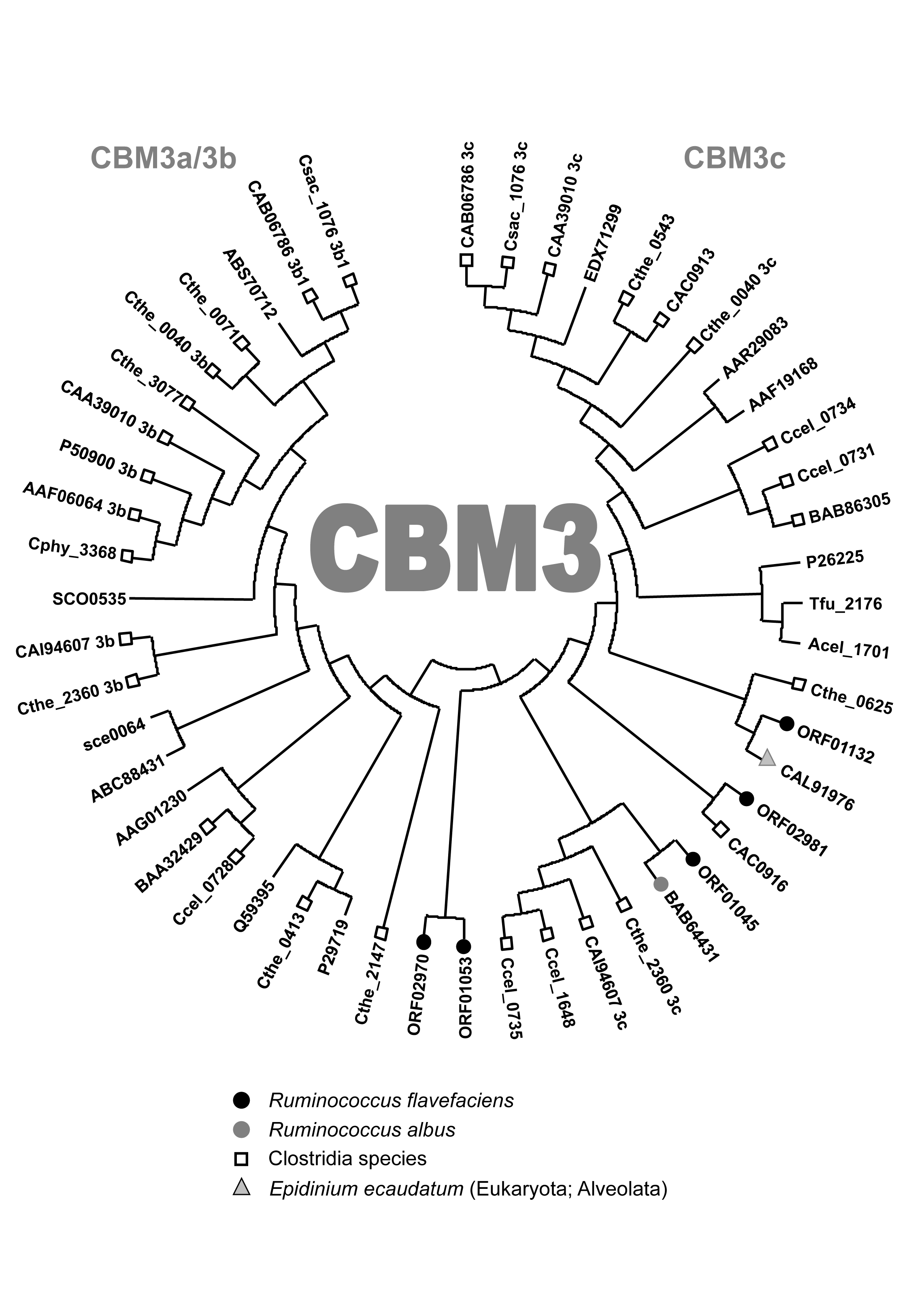

Supplement: Figure S2 — Unrooted dendrogram of putative family 3 carbohydrate-binding modules detected in R. flavefaciens FD-1 compared with those from other organisms. “RfFD-1” refers to R. flavefaciens FD-1, and is followed by ORF designation number assigned by TIGR's Annotation Engine. “Clotm” refers to C. thermocellum, “Rumal” refers to R. albus, and these are followed by the enzyme name. (0.80 MB TIF) [file pone.0006650.s002.tif]

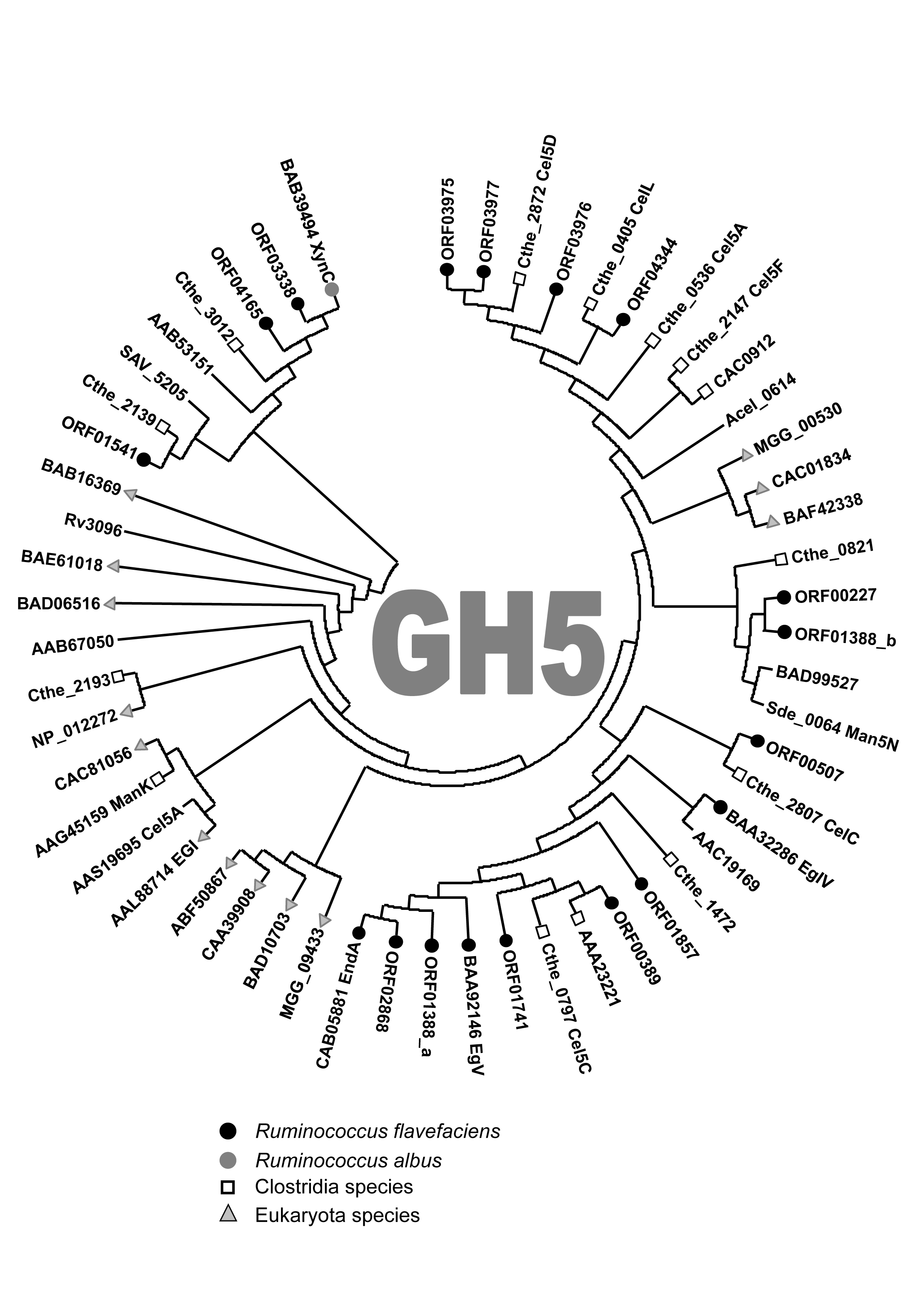

Supplement: Figure S3 — Unrooted dendrogram of glycoside hydrolase family 5 modules detected in R. flavefaciens FD-1 compared with those from other organisms. “Rf” refers to R. flavefaciens, and the ORF number refers to TIGR's Annotation Engine designation. The scale bar indicates the percentage (0.1) of amino acid substitutions. (0.82 MB TIF) [file pone.0006650.s003.tif]

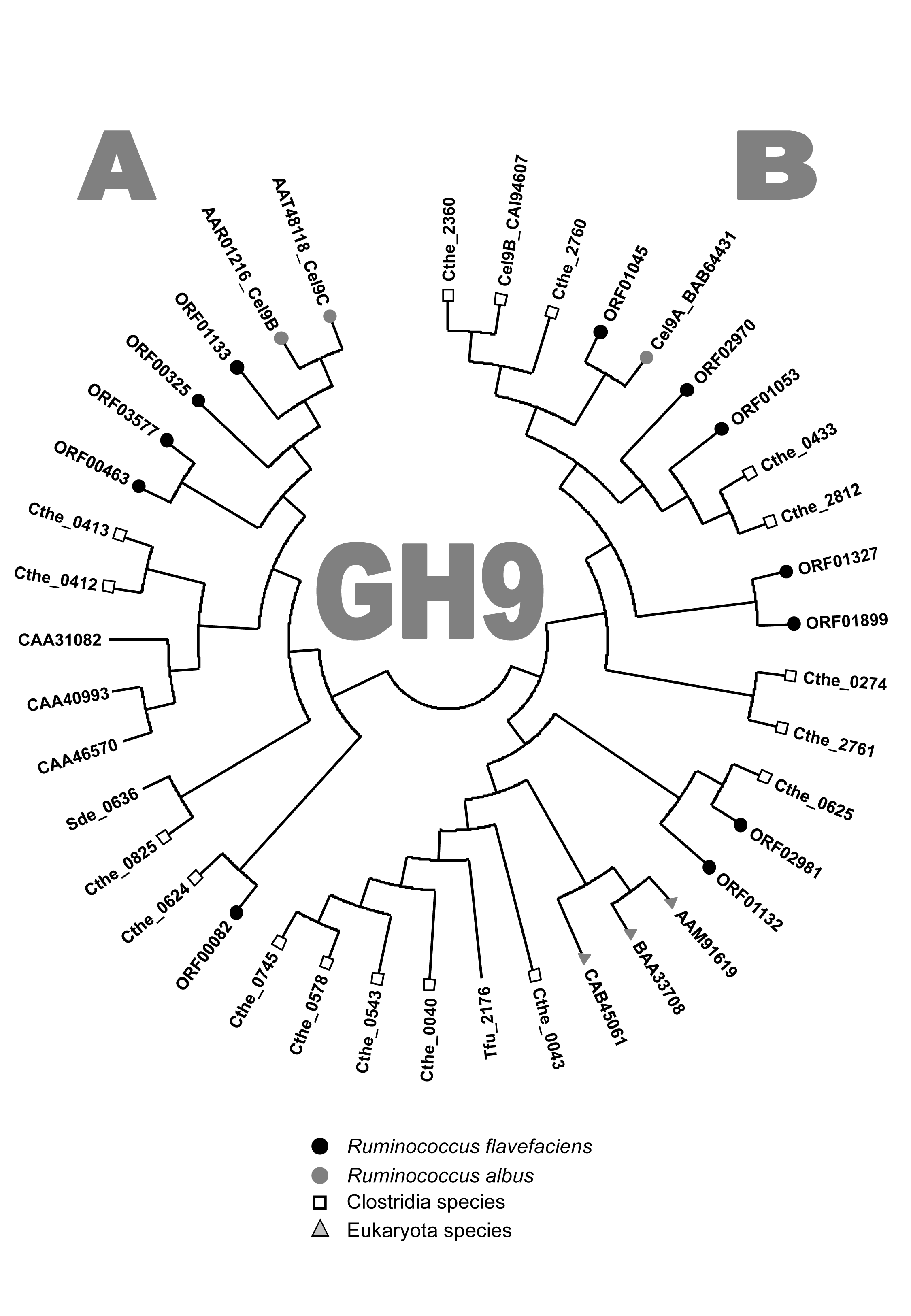

Supplement: Figure S4 — Unrooted dendrogram of glycoside hydrolase family 9 modules detected in R. flavefaciens FD-1 compared with those from other organisms. “Rf” refers to R. flavefaciens, and the ORF number refers to TIGR's Annotation Engine designation. The scale bar indicates the percentage (0.1) of amino acid substitutions. (0.77 MB TIF) [file pone.0006650.s004.tif]
