# Supplementary material for: Diversity and Strain Specificity of Plant Cell Wall Degrading Enzymes Revealed by the Draft Genome of Ruminococcus flavefaciens FD-1
Source: PLoS One. 2009 Aug 14;4(8):e6650. doi: 10.1371/journal.pone.0006650 (PMC2721979; doi:10.1371/journal.pone.0006650)
Supplement: Table S3 — (0.09 MB DOC) [file pone.0006650.s007.doc]

**Table S3.** Proteins used for GH48 phylogeny.

| **Gene/protein name, accession number** | Organism | Enzyme | **Notes and reference*** |
| --- | --- | --- | --- |
| AAC38571 or ExgS | *Clostridium cellulovorans* (Bacteria; Firmicutes; Clostridia) | exoglucanase, contains a dockerin | [1,2] |
| AAN76735 or Cel48A | *Piromyces equi* (Eukaryota; Fungi) | cellulase, contains two CBM10 | [3] |
| AAR01217 or Cel48A | ***Ruminococcus albus* 8** (Bacteria; Firmicutes; Clostridia) | processive endocellulase, contains CBM37 | [4] |
| AAR23324 or Cel48A | *Bacteroides cellulosolvens* DSM 2933 (Bacteria; Firmicutes; Clostridia; **unclassified Ruminococcaceae**) | processive glycoside hydrolase (cellulase) | [5] |
| ABD64772 or Cel48 | *Myxobacter* sp. AL-1 (Bacteria; Deltaproteobacteria) | exocellobiohydrolase | [6] |
| Acel_0617 or YP_872376 | *Acidothermus cellulolyticus* 11B (Actinobacteria) | secreted cellulase, contains CBM2 and CBM3 |  |
| BAA32430 or CelD | *Clostridium josui* (Bacteria; Firmicutes; Clostridia) | exoglucanase, contains a dockerin | [7] |
| BAE94321 or apap2 | *Gastrophysa atrocyanea* (Eukaryota; Metazoa) | Chitinase, an active phase-associated protein II | [8] |
| BL01231 or CelB | *Bacillus licheniformis* ATCC 14580 (Bacteria; Firmicutes) |  |  |
| CAB06786 or CelA | *Anaerocellum thermophilum* DSM 6725 (Bacteria; Firmicutes; Clostridia) | endo−β−1,4-glucanase, contains three CBM3 |  |
| CAC0911 or NP_347547 | *Clostridium acetobutylicum* ATCC 824 (Bacteria; Firmicutes; Clostridia) | processive endoglucanase |  |
| CAD32945 or Cel48C | *Paenibacillus barcinonensis* BP-23 (Bacteria; Firmicutes) | cellulose 1,4−β−cellobiosidase | [9] |
| CAH25542 or Cbh1 | *Otiorhynchus sulcatus* (Eukaryota; Metazoa) | cellulose 1,4−β−cellobiosidase |  |
| Ccel_0729 or CelF | *Clostridium cellulolyticum* H10 (Bacteria; Firmicutes; Clostridia) | processive endocellulase | [10] **(PDB: 1F9D)** |
| Cphy_3368 or YP_001560460 | *Clostridium phytofermentans* ISDg (Bacteria; Firmicutes; Clostridia) | cellulose 1,4−β−cellobiosidase, contains CBM3 |  |
| Csac_1076 or CelA, AAA91086 | *Caldicellulosiruptor saccharolyticus* DSM 8903 (Bacteria; Firmicutes; Clostridia) | endo−β−1,4-glucanase, contains three CBM3 | [11] |
| Cthe_0071 or Cel48B | *Clostridium thermocellum* ATCC 27405 (Bacteria; Firmicutes; Clostridia) | cellulose 1,4−β−cellobiosidase, contains CBM3 |  |
| Cthe_2089 or Cel48S | *C. thermocellum* ATCC 27405 | endo−β−1,4-glucanase (cellobiohydrolase), the major enzymatic component of cellulosome | [12] **(PDB: 1L1Y)** |
| Haur_0295 or YP_001543075 | *Herpetosiphon aurantiacus* ATCC 23779 (Bacteria; Chloroflexi) | secreted cellulase, contains CBM2 |  |
| HCH_02465 or YP_433697 | *Hahella chejuensis* KCTC 2396 (Bacteria; Gammaproteobacteria) | GH48 family protein, contains CBM2 |  |
| ORF03925 | ***Ruminococcus flavefaciens* FD-1** | [SIGN-**GH48**-DOC1] |  |
| P50899 or CbhB or CenE | *Cellulomonas fimi* (Actinobacteria) | 1,4−β−cellobiohydrolase (exocellobiohydrolase) | [13] |
| P50900 or CelY | *Clostridium stercorarium* (Bacteria; Firmicutes; Clostridia) | exo-1,4−β−glucanase (avicelase II) | [14] |
| SCO6546 or NP_630627 | *Streptomyces coelicolor* A3(2) (Actinobacteria) | secreted cellulase |  |
| Tfu_1959 or YP_290015 | *Thermobifida fusca* YX (Actinobacteria) | cellulose 1,4−β−cellobiosidase, contains CBM2 | [15] |

* articles describing a 3D structure of GH5 marked with PDB accession number(s).

**References**

1. Liu CC, Doi RH (1998) Properties of exgS, a gene for a major subunit of the *Clostridium cellulovorans* cellulosome. Gene 211: 39-47.

2. Tamaru Y, Karita S, Ibrahim A, Chan H, Doi RH (2000) A large gene cluster for the *Clostridium cellulovorans* cellulosome. J Bacteriol 182: 5906-5910.

3. Steenbakkers PJ, Ubhayasekera W, Goossen HJ, van Lierop EM, van der Drift C, et al. (2002) An intron-containing glycoside hydrolase family 9 cellulase gene encodes the dominant 90 kDa component of the cellulosome of the anaerobic fungus *Piromyces sp.* strain E2. Biochemical Journal 365: 193-204.

4. Devillard E, Goodheart DE, Karnati SK, Bayer EA, Lamed R, et al. (2004) *Ruminococcus albus* 8 mutants defective in cellulose degradation are deficient in two processive endocellulases, Cel48A and Cel9B, both of which possess a novel modular architecture. Journal of Bacteriology 186: 136-145.

5. Xu Q, Bayer EA, Goldman M, Kenig R, Shoham Y, et al. (2004) Architecture of the *Bacteroides cellulosolvens* cellulosome: description of a cell-surface anchoring scaffoldin and a family-48 cellulase. Journal of Bacteriology 186: 968-977.

6. Avitia CI, Castellanos-Juarez FX, Sanchez E, Tellez-Valencia A, Fajardo-Cavazos P, et al. (2000) Temporal secretion of a multicellulolytic system in *Myxobacter sp*. AL-1. Molecular cloning and heterologous expression of *cel9* encoding a modular endocellulase clustered in an operon with *cel48*, an exocellobiohydrolase gene. European Journal of Biochemistry 267: 7058-7064.

7. Kakiuchi M, Isui A, Suzuki K, Fujino T, Fujino E, et al. (1998) Cloning and DNA sequencing of the genes encoding *Clostridium josui* scaffolding protein CipA and cellulase CelD and identification of their gene products as major components of the cellulosome. J Bacteriol 180: 4303-4308.

8. Fujita K, Shimomura K, Yamamoto K, Yamashita T, Suzuki K (2006) A chitinase structurally related to the glycoside hydrolase family 48 is indispensable for the hormonally induced diapause termination in a beetle. Biochemical and Biophysical Research Communications 345: 502-507.

9. Sanchez MM, Irwin DC, Pastor FI, Wilson DB, Diaz P (2004) Synergistic activity of *Paenibacillus sp.* BP-23 cellobiohydrolase Cel48C in association with the contiguous endoglucanase Cel9B and with endo- or exo-acting glucanases from *Thermobifida fusca*. Biotechnol Bioeng 87: 161-169.

10. Parsiegla G, Juy M, Reverbel-Leroy C, Tardif C, Belaich JP, et al. (1998) The crystal structure of the processive endocellulase CelF of *Clostridium cellulolyticum* in complex with a thiooligosaccharide inhibitor at 2.0 Å resolution. EMBO J 17: 5551-5562.

11. Te'o VS, Saul DJ, Bergquist PL (1995) CelA, another gene coding for a multidomain cellulase from the extreme thermophile *Caldocellum saccharolyticum*. Applied Microbiology and Biotechnology 43: 291-296.

12. Guimaraes BG, Souchon H, Lytle BL, Wu JHD, Alzari PM (2002) The crystal structure and catalytic mechanism of cellobiohydrolase CelS, the major enzymatic component of the *Clostridium thermocellum* cellulosome. Journal of Molecular Biology 320: 587-596.

13. Shen H, Tomme P, Meinke A, Gilkes NR, Kilburn DG, et al. (1994) Stereochemical course of hydrolysis catalysed by Cellulomonas fimi CenE, a member of a new family of beta-1,4-glucanases. Biochem Biophys Res Commun 199: 1223-1228.

14. Bronnenmeier K, Kundt K, Riedel K, Schwarz W, Staudenbauer W (1997) Structure of the *Clostridium stercorarium* gene *cel*Y encoding the exo-1,4-β-glucanase Avicelase II. Microbiology 143: 891-898.

15. Irwin DC, Zhang S, Wilson DB (2000) Cloning, expression and characterization of a family 48 exocellulase, Cel48A, from Thermobifida fusca. Eur J Biochem 267: 4988-4997.
