# Supplementary material for: Diversity and Strain Specificity of Plant Cell Wall Degrading Enzymes Revealed by the Draft Genome of Ruminococcus flavefaciens FD-1
Source: PLoS One. 2009 Aug 14;4(8):e6650. doi: 10.1371/journal.pone.0006650 (PMC2721979; doi:10.1371/journal.pone.0006650)
Supplement: Table S5 — (0.11 MB DOC) [file pone.0006650.s009.doc]

**Table S5.** Proteins used for CBM3 Phylogeny.

| **Gene/protein name** | **Organism** | **Notes and reference*** |
| --- | --- | --- |
| AAF06064 or CipV | *Acetivibrio cellulolyticus* | Scaffoldin-based CBM3b; [1] |
| AAF19168 or Cel9 | *Myxobacter* sp. AL-1 (deltaproteobacteria) | CBM3c; [2] |
| AAG01230 or ScaA/CipBc | *Bacteroides cellulosolvens* | Scaffoldin-based CBM3b; [3,4] |
| AAR29083 or BL01232 or Cel9A | *Bacillus licheniformis* GXN151 | CBM3c; [5] |
| ABS70712 or CelA/BglC | *Bacillus subtilis* BS-2 |  |
| ABC88431 or Cel44C | *Paenibacillus polymyxa* GS01 | [6] |
| Acel_1701 | *Acidothermus cellulolyticus* 11B |  |
| BAA32429 or CipJ | *Clostridium josui* | Scaffoldin-based CBM3a; [7] |
| BAB64431 or Cel9A or EgVI | ***Ruminococcus albus* strain F-40** | CBM3c; [8] |
| BAB86305 or CbpA | *Eubacterium cellulosolvens* | [9] |
| CAA39010 or CelZ | *Clost. stercorarium* NCIB 11745 | CBM3b and CBM3c; [10] |
| CAB06786 or CelA | *Anaerocellum thermophilum* | CBM3b and CBM3c |
| CAC0913 or NP_347549 or Cel9A-G | *Clost. acetobutylicum* ATCC 824 | CBM3c |
| CAC0916 or NP_347552 or Cel9B-G | *Clost. acetobutylicum* ATCC 824 | CBM3c |
| CAI94607 or Cel9B | *Acetivibrio cellulolyticus* CD2 | CBM3b and CBM3c; [11] |
| CAL91976 | *Epidinium ecaudatum* (Eukaryota; Alveolata) |  |
| Ccel_0728 or YP_002505087 or CipC | *Clost. cellulolyticum* H10 | Scaffoldin-based CBM3a; [12] **PDB 1G43)** |
| Ccel_0731 or YP_002505090 or Cel9G | *Clost. cellulolyticum* H10 | [13]**(PDB 1G87)** |
| Ccel_0734 or YP_002505093 or Cel9-H, AAG45157 | *Clost. cellulolyticum* H10 | CBM3c; Belaich *et al*., 2000, unpublished |
| Ccel_0735 or YP_002505094 or Cel9-J, AAG45158 | *Clost. cellulolyticum* H10 | CBM3c; Belaich *et al*., 2000, unpublished |
| Ccel_1648 or YP_002505979 | *Clost. cellulolyticum* H10 |  |
| Cphy_3368 or CelI | *Clost. phytofermentans* |  |
| Csac_1076 or Cel9A, AAA91086, P22534 | *Caldicellulosiruptor saccharolyticus* | CBM3b and CBM3c;[14];[15] |
| Cthe_0040 or Cel9I; Q02934 | *Clost. thermocellum* ATCC 27405 | CBM3b and CBM3c; [5] |
| Cthe_0071 or Cel48B (Cel48Y) | *Clost. thermocellum* ATCC 27405 | CBM3b; [16] (strain F7) |
| Cthe_0413 or YP_001036844 or CbhA | *Clost. thermocellum* ATCC 27405 | [17](strain F7) |
| Cthe_0543 or YP_001036972 or Cel9F, P26224 | *Clost. thermocellum* ATCC 27405 | CBM3c; [18] |
| Cthe_0625 or YP_001037053 or Cel9Q | *Clost. thermocellum* ATCC 27405 | CBM3c |
| Cthe_2147 or Cel5O | *Clost. thermocellum* ATCC 27405 |  |
| Cthe_2360 or Cel9U | *Clost. thermocellum* ATCC 27405 | CBM3b and CBM3c; [11] |
| Cthe_3077 or YP_001039466 or CipA | *Clost. thermocellum* ATCC 27405 | Scaffoldin-based CBM3a; [19,20,21]**(PDB 1NBC)** |
| EDX71299 | *Microcoleus chthonoplastes* PCC 7420 (cyanobacteria) |  |
| ORF01045 GH9 | ***Ruminococcus flavefaciens* strain FD-1** |  |
| ORF01053 GH9 | ***Ruminococcus flavefaciens* strain FD-1** |
| ORF01132 GH9 | ***Ruminococcus flavefaciens* strain FD-1** |
| ORF02970 GH9 | ***Ruminococcus flavefaciens* strain FD-1** |
| ORF02981 GH9 | ***Ruminococcus flavefaciens* strain FD-1** |
| P29719 or CelA | *Paenibacillus lautus* | [22] |
| P26225 or CenB | *Cellulomonas fimi* (actinobacteria) | [23,24] |
| P50900 or CelY | *Clost. stercorarium* | CBM3b; [25] |
| Q59395 or CelV1 | *Pectobacterium* *carotovorum* (gammaproteobacteria) | [26] |
| SCO0535 | *Streptomyces coelicolor* A3(2) (actinobacteria) |  |
| sce0064 | *Sorangium cellulosum* (deltaproteobacteria) |  |
| Tfu_2176 or E4 (Cel9A) | *Thermobifida fusca* YX | CBM3c; [27] **(PDB 1JS4)** |

* articles describing a 3D structure of CBM marked with PDB accession number(s).

**References**

1. Ding S-Y, Bayer EA, Steiner D, Shoham Y, Lamed R (1999) A novel cellulosomal scaffoldin from *Acetivibrio cellulolyticus* that contains a family-9 glycosyl hydrolase. Journal of Bacteriology 181: 6720-6729.

2. Avitia CI, Castellanos-Juarez FX, Sanchez E, Tellez-Valencia A, Fajardo-Cavazos P, et al. (2000) Temporal secretion of a multicellulolytic system in *Myxobacter sp*. AL-1. Molecular cloning and heterologous expression of *cel9* encoding a modular endocellulase clustered in an operon with *cel48*, an exocellobiohydrolase gene. European Journal of Biochemistry 267: 7058-7064.

3. Ding S-Y, Bayer EA, Steiner D, Shoham Y, Lamed R (2000) A scaffoldin of the *Bacteroides cellulosolvens* cellulosome that contains 11 type II cohesins. Journal of Bacteriology 182: 4915-4925.

4. Xu Q, Bayer EA, Goldman M, Kenig R, Shoham Y, et al. (2004) Architecture of the *Bacteroides cellulosolvens* cellulosome: description of a cell-surface anchoring scaffoldin and a family-48 cellulase. Journal of Bacteriology 186: 968-977.

5. Liu Y, Zhang J, Liu Q, Zhang C, Ma Q (2004) Molecular cloning of novel cellulase genes *cel9A* and *cel12A* from *Bacillus licheniformis* GXN151 and synergism of their encoded polypeptides. Current Microbiology 49: 234-238.

6. Cho KM, Hong SY, Lee SM, Kim YH, Kahng G, et al. (2006) A *cel44C-man26A* gene of endophytic *Paenibacillus polymyxa* GS01 has multi-glycosyl hydrolases in two catalytic domains. Applied Microbiology and Biotechnology 73: 618-630.

7. Kakiuchi M, Isui A, Suzuki K, Fujino T, Fujino E, et al. (1998) Cloning and DNA sequencing of the genes encoding *Clostridium josui* scaffolding protein CipA and cellulase CelD and identification of their gene products as major components of the cellulosome. J Bacteriol 180: 4303-4308.

8. Taguchi H, Hagiwara D, Genma T, Karita S, Kimura T, et al. (2004) Cloning of the *Ruminococcus albus* *cel5D* and *cel9A* genes encoding dockerin module-containing endoglucanases and expression of *cel5D* in *Escherichia coli*. Biosci Biotechnol Biochem 68: 1557-1564.

9. Toyoda A, Minato H (2002) Cloning, nucleotide sequence and expression of the gene encoding the cellulose-binding protein A (CBPA) of *Eubacterium cellulosolvens* 5. FEMS Microbiology Letters 207: 141-146.

10. Jauris S, Rücknagel KP, Schwarz WH, Kratzsch P, Bronnenmeier K, et al. (1990) Sequence analysis of the *Clostridium stercorarium* *celZ* gene encoding a thermoactive cellulase (AvicelaseI). Molecular and General Genetics 223: 258-267.

11. Jindou S, Xu Q, Kenig R, Shoham Y, Bayer EA, et al. (2006) Novel architectural theme of family-9 glycoside hydrolases identified in cellulosomal enzymes of *Acetivibrio cellulolyticus* and *Clostridium thermocellum*. FEMS Microbiology Letters 254: 308-316.

12. Shimon LJW, Pages S, Belaich A, Belaich JP, Bayer EA, et al. (2000) Structure of a family IIIa scaffoldin CBD from the cellulosome of *Clostridium cellulolyticum* at 2.2 Å resolution. Acta Crystallogr D Biol Crystallogr 56: 1560-1568.

13. Mandelman D, Belaich A, Belaich JP, Aghajari N, Driguez H, et al. (2003) X-Ray crystal structure of the multidomain endoglucanase Cel9G from *Clostridium cellulolyticum* complexed with natural and synthetic cello-oligosaccharides. J Bacteriol 185: 4127-4135.

14. Luthi E, Jasmat NB, Grayling RA, Love DR, Bergquist PL (1991) Cloning, sequence analysis, and expression in Escherichia coli of a gene coding for a beta-mannanase from the extremely thermophilic bacterium "Caldocellum saccharolyticum". Appl Environ Microbiol 57: 694-700.

15. Te'o VS, Saul DJ, Bergquist PL (1995) CelA, another gene coding for a multidomain cellulase from the extreme thermophile *Caldocellum saccharolyticum*. Applied Microbiology and Biotechnology 43: 291-296.

16. Berger E, Zhang D, Zverlov VV, Schwarz WH (2007) Two noncellulosomal cellulases of *Clostridium thermocellum*, Cel9I and Cel48Y, hydrolyse crystalline cellulose synergistically. FEMS Microbiology Letters 268: 194-201.

17. Zverlov VV, Velikodvorskaya GV, Schwarz WH, Bronnenmeier K, Kellermann J, et al. (1998) Multidomain structure and cellulosomal localization of the *Clostridium thermocellum* cellobiohydrolase CbhA. J Bacteriol 180: 3091-3099.

18. Shen H, Tomme P, Meinke A, Gilkes NR, Kilburn DG, et al. (1994) Stereochemical course of hydrolysis catalysed by Cellulomonas fimi CenE, a member of a new family of beta-1,4-glucanases. Biochem Biophys Res Commun 199: 1223-1228.

19. Lamed R, Tormo J, Chirino AJ, Morag E, Bayer EA (1994) Crystallization and preliminary X-ray analysis of the major cellulose-binding domain of the cellulosome from *Clostridium thermocellum*. Journal of Molecular Biology 244: 236-237.

20. Morag E, Lapidot A, Govorko D, Lamed R, Wilchek M, et al. (1995) Expression, purification and characterization of the cellulose-binding domain of the scaffoldin subunit from the cellulosome of *Clostridium thermocellum*. Applied and Environmental Microbiology 61: 1980-1986.

21. Tormo J, Lamed R, Chirino AJ, Morag E, Bayer EA, et al. (1996) Crystal structure of a bacterial family-III cellulose-binding domain: A general mechanism for attachment to cellulose. EMBO J 15: 5739-5751.

22. Hansen CK, Diderichsen B, Joergensen PL (1992) *celA* from *Bacillus lautus* PL236 encodes a novel cellulose-binding endo-β-1,4-glucanase. Journal of Bacteriology 174: 3522-3531.

23. Meinke A, Braun C, Gilkes NR, Kilburn DG, Miller RC, et al. (1991) Unusual sequence organization in CenB, an inverting endoglucanase from Cellulomonas fimi. J Bacteriol 173: 308-314.

24. Meinke A, Gilkes NR, Kilburn DG, Miller RCJ, Warren RAJ (1991) Multiple domains in endoglucanase B (CenB) from *Cellulomonas fimi:* Functions and relatedness to domains in other polypeptides. Journal of Bacteriology 173: 7126-7135.

25. Bronnenmeier K, Kundt K, Riedel K, Schwarz W, Staudenbauer W (1997) Structure of the *Clostridium stercorarium* gene *cel*Y encoding the exo-1,4-β-glucanase Avicelase II. Microbiology 143: 891-898.

26. Mae A, Heikinheimo R, Palva ET (1995) Structure and regulation of the *Erwinia carotovora* subspecies *carotovora* SCC3193 cellulase gene *celV1* and the role of cellulase in phytopathogenicity. Molecular and General Genetics 247: 17-26.

27. Sakon J, Irwin D, Wilson DB, Karplus PA (1997) Structure and mechanism of endo/exocellulase E4 from Thermomonospora fusca. Nature Struct Biol 4: 810-818.
