# Supplementary material for: Diversity and Strain Specificity of Plant Cell Wall Degrading Enzymes Revealed by the Draft Genome of Ruminococcus flavefaciens FD-1
Source: PLoS One. 2009 Aug 14;4(8):e6650. doi: 10.1371/journal.pone.0006650 (PMC2721979; doi:10.1371/journal.pone.0006650)
Supplement: Table S6 — (0.14 MB DOC) [file pone.0006650.s010.doc]

**Table S6.** Protein sequences used for GH5 phylogeny

| **Gene/protein name, accession number** | Organism | Enzyme | **Notes and reference*** |
| --- | --- | --- | --- |
| AAA23221 or Cel5A | *Clostridium cellulolyticum* H10 (Bacteria; Firmicutes) | endo−β−1,4-glucanase **(GH5_4)** | [1,2] **(PDB: 1EDG)** |
| AAB53151 | *Erwinia chrysanthemi* D1 (Bacteria; Gammaproteobacteria) | Xylanase **(GH5_8)** | [3,4] **(PDB: 1NOF)** |
| AAB67050 | *Rhodococcus* sp. strain M-777 (Actinobacteria) | Endoglycoceramidase **(GH5_18)** | [5] |
| AAC19169 or Cel5A | *Bacillus agaradhaerens* ATCC (Bacteria; Firmicutes) | endo−β−1,4-glucanase **(GH5_2)** |  |
| AAG45159 or Man5K | *C. cellulolyticum* H10 (Bacteria; Firmicutes) | Mannanase **(GH5_20)** |  |
| AAL88714 or EGI | *Thermoascus aurantiacus* IFO 9748 (Eukaryota; Fungi) | endo−β−1,4-glucanase **(GH5_5)** | [6] |
| AAS19695 or Man5A | *Cellvibrio mixtus* (Bacteria; Gammaproteobacteria) | β−mannosidase **(GH5_6)** | [7] **(PDB: 1UUQ)** |
| ABF50867 or AN3777-2 | *Emericella nidulans* FGSC A4 (Eukaryota; Fungi) | β−1,6-glucanase **(GH5_15)** | [8] |
| Acel_0614 or AAA75477 | *Acidothermus cellulolyticus* 11B (Actinobacteria) | endo−β−1,4-glucanase **(GH5_1)** | [9] **(PDB: 1ECE; 1VRX)** |
| BAA32286 or Eg IV | *Ruminococcus albus* F-40 (Bacteria; Firmicutes) | endo−β−1,4-glucanase | [10] |
| BAA92146 or EgV | *R. albus* F-40 (Bacteria; Firmicutes) | endo−β−1,4-glucanase | [11] |
| BAB16369 | *Cyanea nozakii* (Eukaryota; jellyfish) | Endoglycoceramidase **(GH5_19)** | [12] |
| BAB39494 or XynC | ***Ruminococcus albus* strain 7** (Bacteria; Firmicutes) | Xylanase **(Pfam02055: GH30 family)** | [13] |
| BAD06516 | *Paecilomyces lilacinus* (Eukaryota; Fungi) | β−1,3-mannanase **(GH5_24)** | [14] |
| BAD10703 or OsJ_026603 | *Oryza sativa* Japonica Group (Eukaryota; Viridiplantae) | β−1,3- glucanase **(GH5_10)** |  |
| BAD99527 or Amn5A | *Bacillus* sp. JAMB−602 (Bacteria; Firmicutes) | β−mannanase **(GH5_7)** |  |
| BAE61018 | *Aspergillus oryzae* RIB 40 (Eukaryota; Fungi) | Cellulase **(GH5_22)** |  |
| BAF42338 or Fogal1 | *Fusarium oxysporum* 12s (Eukaryota; Fungi) | β−1,6-galactanase **(GH5_16)** | [15] |
| CAA39908 or XP_721488 | *Candida albicans* ATCC 10261 (Eukaryota; Fungi) | exo−β−1,3-glucosidase **(GH5_9)** | [16] |
| CAB05881 or EndA | ***Ruminococcus flavefaciens* strain 17** (Bacteria; Firmicutes) | endo−β−1,4-glucanase | [17] |
| CAC01834 or F5E19_40 | *Arabidopsis thaliana* (Eukaryota; Viridiplantae) | Endoglucanase **(GH5_11)** |  |
| CAC0912 or NP_347548 | *C. acetobutylicum* ATCC 824 (Bacteria; Firmicutes) | non-processive endoglucanase | [18] |
| CAC81056 | *Mytilus edulis* (Eukaryota; bivalves; edible mussel) | β−1,4-mannanase **(GH5_21)** |  |
| Cthe_0405 or CelL | *Clostridium thermocellum* ATCC 27405 (Bacteria; Firmicutes) | endo−β−1,4-glucanase L |  |
| Cthe_0536 or Cel5A or CelB, or EgB |  | endo−β−1,4-glucanase B | [19,20] **(PDB: 1CEC)** |
| Cthe_0797 or Cel5C or CelE |  | endo−β−1,4-glucanase E |  |
| Cthe_0821 |  |  |  |
| Cthe_1472 |  |  |  |
| Cthe_2139 |  | **Pfam02055: GH30 family** |  |
| Cthe_2147 or Cel5F or CelO |  | 1,4−β−cellobiohydrolase |  |
| Cthe_2193 |  |  |  |
| Cthe_2807 or CelC |  | endo−β−1,4-glucanase C |  |
| Cthe_2872 or Cel5D or CelG |  | endo−β−1,4-glucanase G |  |
| Cthe_3012 | *C. thermocellum* ATCC 27405 (Bacteria; Firmicutes) | **Pfam02055: GH30 family** |  |
| MGG_00530 or A4REX8_MAGGR | *Magnaporthe grisea* (Eukaryota; Fungi) | Cellulase **(GH5_23)** |  |
| MGG_09433 or A4R3V8_MAGGR | *M. grisea* (Eukaryota; Fungi) | Cellulase **(GH5_17)** |  |
| NP_012272 or YIR007W | *Saccharomyces cerevisiae* S288C (Eukaryota; Fungi) | Cellulase **(GH5_12)**. A GFP-fusion protein localizes to the cytoplasm |  |
| ORF00227 | ***Ruminococcus flavefaciens* strain FD-1** (Bacteria; Firmicutes) | [SIGN-CBM35-**GH5**-DOC] |  |
| ORF00389 |  | [SIGN-**GH5**-UNK-DOC] |  |
| ORF00507 |  | [SIGN-**GH5**] |  |
| ORF01388_a, |  | [SIGN-**GH5**-UNK-**GH5**-DOC] |  |
| ORF01388_b |  | [SIGN-**GH5**-UNK-**GH5**-DOC] |  |
| ORF01541 |  | [SIGN-**GH5**-CBM32-CBM32-DOC]; Pfam02055: GH30 family |  |
| ORF01741 |  | [SIGN-UNK-**GH5**] |  |
| ORF01857 |  | [SIGN-**GH5**-DOC] |  |
| ORF02868 |  | [SIGN-**GH5**-UNK-DOC] |  |
| ORF03338 |  | [SIGN-**GH5**-UNK-CBM22-DOC]; Pfam02055: GH30 family |  |
| ORF03975 |  | [SIGN-**GH5**-UNK-DOC] |  |
| ORF03976 |  | [SIGN-**GH5**-DOC] |  |
| ORF03977 |  | [SIGN-**GH5**-DOC] |  |
| ORF04165 |  | [SIGN-**GH5**-UNK-CBM22-DOC-UNK]; **Pfam02055: GH30 family** |  |
| ORF04344 | ***R. flavefaciens* strain FD-1** (Bacteria; Firmicutes) | [SIGN-**GH5**-UNK-DOC] |  |
| Rv3096 or NP_217612 | *Mycobacterium tuberculosis* H37Rv (Actinobacteria) | GH5_13 |  |
| SAV_5205 or BAC72917 | *Streptomyces avermitilis* MA-4680 (Actinobacteria) | **GH5_14** |  |
| Sde_0064 or Man5N | *Saccharophagus degradans* 2-40 (Bacteria; Gammaproteobacteria) |  |  |

* articles describing a 3D structure of GH5 marked with PDB accession number(s).

**References**

1. Faure E, Belaich A, Bagnara C, Gaudin C, Belaich J-P (1989) Sequence analysis of the *Clostridium cellulolyticum* endoglucanase-A-encoding gene *celCCA*. Gene 84: 39-46.

2. Ducros V, Czjzek M, Belaich A, Gaudin C, Fierobe H-P, et al. (1995) Crystal structure of the catalytic domain of a bacterial cellulase belonging to family 5. Structure 3: 939-949.

3. Keen NT, Boyd C, Henrissat B (1996) Cloning and characterization of a xylanase gene from corn strains of *Erwinia chrysanthemi*. Mol Plant Microbe Interact 9: 651-657.

4. Larson SB, Day J, Barba de la Rosa AP, Keen NT, McPherson A (2003) First crystallographic structure of a xylanase from glycoside hydrolase family 5: implications for catalysis. Biochemistry 42: 8411-8422.

5. Izu H, Izumi Y, Kurome Y, Sano M, Kondo A, et al. (1997) Molecular cloning, expression, and sequence analysis of the endoglycoceramidase II gene from *Rhodococcus species* strain M-777. Journal of Biological Chemistry 272: 19846-19850.

6. Hong J, Tamaki H, Yamamoto K, Kumagai H (2003) Cloning of a gene encoding a thermo-stable endo-beta-1,4 glucanase from *Thermoascus aurantiacus* and its expression in yeast. Biotechnology Letters 25: 657-661.

7. Dias FM, Vincent F, Pell G, Prates JA, Centeno MS, et al. (2004) Insights into the molecular determinants of substrate specificity in glycoside hydrolase family 5 revealed by the crystal structure and kinetics of *Cellvibrio mixtus* mannosidase 5A. Journal of Biological Chemistry 279: 25517-25526.

8. Bauer S, Vasu P, Persson S, Mort AJ, Somerville CR (2006) Development and application of a suite of polysaccharide-degrading enzymes for analyzing plant cell walls. Proceedings of the National Academy of Sciences USA 103: 11417-11422.

9. Sakon J, Adney W, Himmel M, Thomas S, Karplus P (1996) Crystal structure of thermostable family 5 endocellulase E1 from *Acidothermus cellulolyticus* in complex with cellotetraose. Biochemistry 35: 10648-10660.

10. Karita S, Kimura T, Sakka K, Ohmiya K (1997) Purification of the *Ruminococcus albus* endoglucanase IV using a cellulose-binding domain as an affinity tag. Journal of Fermentation and Bioengineering 84: 354-357.

11. Ohara H, Noguchi J, Karita S, Kimura T, Sakka K, et al. (2000) Sequence of egV and properties of EgV, a *Ruminococcus albus* endoglucanase containing a dockerin domain. Biosci Biotechnol Biochem 64: 80-88.

12. Horibata Y, Okino N, Ichinose S, Omori A, Ito M (2000) Purification, characterization, and cDNA cloning of a novel acidic endoglycoceramidase from the jellyfish, *Cyanea nozakii*. Journal of Biological Chemistry 275: 31297-31304.

13. Nakamura M, Nagamine T, Takenaka A, Aminov RI, Ogata K, et al. (2002) Molecular cloning, nucleotide sequence and characteristics of a xylanase gene (*xynA*) from *Ruminococcus albus* 7. Animal Science Journal 73: 347-352.

14. Sugino H, Furuichi S, Murao S, Arai M, Fujii T (2004) Molecular characterization of a Rhodotorula-lytic enzyme from

*Paecilomyces lilacinus* having beta-1,3-mannanase activity. Bioscience, Biotechnology, and Biochemistry 68: 757-760.

15. Sakamoto T, Taniguchi Y, Suzuki S, Ihara H, Kawasaki H (2007) Characterization of {beta}-1,6-galactanase of *Fusarium oxysporum*, an enzyme that hydrolyzes larch wood arabinogalactan. Applied and Environmental Microbiology 73: 3109-3112.

16. Chambers RS, Broughton MJ, Cannon RD, Carne A, Emerson GW, et al. (1993) An exo-beta-(1,3)-glucanase of *Candida albicans*: purification of

the enzyme and molecular cloning of the gene. Journal of General Microbiology 139: 325-334.

17. Kirby J, Martin JC, Daniel AS, Flint HJ (1997) Dockerin-like sequences in cellulases and xylanases from the rumen cellulolytic bacterium *Ruminococcus flavefaciens*. FEMS Microbiology Letters 149: 213-219.

18. Nolling J, Breton G, Omelchenko MV, Makarova KS, Zeng Q, et al. (2001) Genome sequence and comparative analysis of the solvent-producing bacterium *Clostridium acetobutylicum*. Journal of Bacteriology 183: 4823-4838.

19. Dominguez R, Souchon H, Spinelli S, Dauter Z, Wilson KS, et al. (1995) A common protein fold and similar active site in two distinct families of β-glycanases. Nature Structural Biology 2: 569-576.

20. Grepinet O, Beguin P (1986) Sequence of the cellulase gene of Clostridium thermocellum coding for endoglucanase B. Nucleic Acids Res 14: 1791-1799.
