# Supplementary material for: Diversity and Strain Specificity of Plant Cell Wall Degrading Enzymes Revealed by the Draft Genome of Ruminococcus flavefaciens FD-1
Source: PLoS One. 2009 Aug 14;4(8):e6650. doi: 10.1371/journal.pone.0006650 (PMC2721979; doi:10.1371/journal.pone.0006650)
Supplement: Table S7 — (0.10 MB DOC) [file pone.0006650.s011.doc]

**Table S7.** Protein sequences used for GH9 phylogeny

| **Gene/protein name, accession number** | **Organism** | **Enzyme** | Notes and reference* |
| --- | --- | --- | --- |
| AAM91619 or At4g11050 | *Arabidopsis thaliana* (Eukaryota; Viridiplantae) | putative glucanase **(GH9_6)**, contains CBM2 |  |
| AAR01216 or Cel9B | ***Ruminococcus albus* 8** (Bacteria; Firmicutes; Clostridia) | processive endocellulase, a theme D group of GH9 family | [1] |
| AAT48118 or Cel9C | ***R. albus* 8** | processive endocellulase, contains CBM37 | [2] |
| BAA33708 (gene NtEG) | termite *Nasutitermes takasagoensis* (Eukaryota; Metazoa) | endo-β−1,4-glucanase **(GH9_5)** | [3] |
| BAB64431 or Cel9A, | ***Ruminococcus albus* F-40** (Bacteria; Firmicutes; Clostridia) | cellulase VI | [4] |
| CAA31082 or CelA | *Cellvibrio japonicus* [formerly *Pseudomonas fluorescens* subsp. cellulosa] (Bacteria; Gammaproteobacteria) | endo-β−1,4-glucanase, contains CBM2 and CBM10 | [5] |
| CAA40993 or CenC | *Cellulomonas fimi* ATCC 484 (Actinobacteria) | Endoglucanase, contains two CBM_4_9 | [6] |
| CAA46570 or Cel1 | *Streptomyces retuculi* (Actinobacteria) | secreted endoglucanase (avicelase, cellulase), contains CBM_4_9 | [7] |
| CAB45061 or At4g24260 | *A. thaliana* (Eukaryota; Viridiplantae) | endo-β−1,4-glucanase **(GH9_4)** | Bevan *et al*., 1999; unpublished |
| CAI94607 or Cel9B | *Acetivibrio cellulolyticus* CD2 (Bacteria; Firmicutes; Clostridia) | cellulosome glycoside hydrolase, contains two CBM3 | [8] |
| Cthe_0040 or Cel9I | *Clostridium thermocellum* ATCC 27405 (Bacteria; Firmicutes; Clostridia) | endo-β−1,4-glucanase |  |
| Cthe_0043 or Cel9N |  | endo-β−1,4-glucanase |  |
| Cthe_0274 or Cel9P |  |  |  |
| Cthe_0412 or Cel9K |  |  |  |
| Cthe_0413 or Cbh9A, YP_001036844 |  | **GH9_1** |  |
| Cthe_0433 |  |  |  |
| Cthe_0543 or Cel9F |  |  |  |
| Cthe_0578 or Cel9R |  |  |  |
| Cthe_0624 or Cel9J (+Cel44A) |  |  |  |
| Cthe_0625 or Cel9Q |  |  |  |
| Cthe_0745 or Cel9W |  |  |  |
| Cthe_0825 or YP_001037253 |  | **GH9_2** |  |
| Cthe_2360 or Cel9U |  |  |  |
| Cthe_2760 or Cel9V |  |  |  |
| Cthe_2761 |  |  |  |
| Cthe_2812 or Cel9T | *C. thermocellum* ATCC 27405 | Endoglucanase |  |
| ORF00082 | ***Ruminococcus flavefaciens* FD-1** (Bacteria; Firmicutes; Clostridia) | [SIGN-CBM4-**GH9**-DOC1] |  |
| ORF00325 |  | [SIGN-CBM4-**GH9**-UNK-TM] |  |
| ORF00463 |  | [SIGN-CBM4-**GH9**-DOC1] |  |
| ORF01045 |  | [SIGN-**GH9**-CBM3-DOC1] |  |
| ORF01053 |  | [SIGN-**GH9**-CBM3-UNK-DOC1] |  |
| ORF01132 |  | [SIGN-**GH9**-CBM3-DOC1] |  |
| ORF01133 |  | [SIGN-CBM4-**GH9**-DOC1] |  |
| ORF01327 |  | [SIGN-**GH9**-UNK-DOC1] |  |
| ORF01899 or CelD, AAA17731 | ***R. flavefaciens* FD-1** | beta-glucanase [UNK-TM-UNK-**GH9**-UNK-SORT] | Vercoe and White, 1992; unpublished |
| ORF02970 |  | [SIGN-**GH9**-CBM3-UNK] |  |
| ORF02981 |  | [SIGN-**GH9**-CBM3-DOC1] |  |
| ORF03577 | ***R. flavefaciens* FD-1** | [SIGN-CBM4-**GH9**-DOC1] |  |
| Sde_0636 or ABD79898 | *Saccharophagus degradans* 2-40 (Bacteria; Gammaproteobacteria) | endo-β−1,4-glucanase **(GH9_2)** |  |
| Tfu_2176 or E4 (CelE), AAB42155 | *Thermobifida fusca* YX (Actinobacteria) | endo-β−1,4-glucanase **(GH9_3)**, contains CBM2 and CBM3; has both endocellulase and exocellulase activity | [9] |

* articles describing a 3D structure of GH9 marked with PDB accession number(s).

**References**

1. Devillard E, Goodheart DE, Karnati SK, Bayer EA, Lamed R, et al. (2004) *Ruminococcus albus* 8 mutants defective in cellulose degradation are deficient in two processive endocellulases, Cel48A and Cel9B, both of which possess a novel modular architecture. Journal of Bacteriology 186: 136-145.

2. Xu Q, Bayer EA, Goldman M, Kenig R, Shoham Y, et al. (2004) Architecture of the *Bacteroides cellulosolvens* cellulosome: description of a cell-surface anchoring scaffoldin and a family-48 cellulase. Journal of Bacteriology 186: 968-977.

3. Tokuda G, Lo N, Watanabe H, Slaytor M, Matsumoto T, et al. (1999) Metazoan cellulase genes from termites: intron/exon structures and sites of expression. Biochim Biophys Acta 1447: 146-159.

4. Taguchi H, Hagiwara D, Genma T, Karita S, Kimura T, et al. (2004) Cloning of the *Ruminococcus albus* *cel5D* and *cel9A* genes encoding dockerin module-containing endoglucanases and expression of *cel5D* in *Escherichia coli*. Biosci Biotechnol Biochem 68: 1557-1564.

5. Gilbert HJ, Sullivan DA, Jenkins G, Kellett LE, Minton NP, et al. (1988) Molecular cloning of multiple xylanase genes from Pseudomonas fluorescens subsp. cellulosa. J Gen Microbiol 134: 3239-3247.

6. Coutinho JB, Moser B, Kilburn DG, Warren RA, Miller RC (1991) Nucleotide sequence of the endoglucanase C gene (cenC) of Cellulomonas fimi, its high-level expression in Escherichia coli, and characterization of its products. Mol Microbiol 5: 1221-1233.

7. Walter S, Schrempf H (1995) Studies of *Streptomyces reticuli cel-1* (cellulase) gene expression in *Streptomyces* strains*, Escherichia coli, and Bacillus subtilis*. Applied and Environmental Microbiology 61: 487-494.

8. Jindou S, Xu Q, Kenig R, Shoham Y, Bayer EA, et al. (2006) Novel architectural theme of family-9 glycoside hydrolases identified in cellulosomal enzymes of *Acetivibrio cellulolyticus* and *Clostridium thermocellum*. FEMS Microbiology Letters 254: 308-316.

9. Lin ES, Wilson DB (1988) Identification of a celE-binding protein and its potential role in induction of the celE gene in Thermomonospora fusca. J Bacteriol 170: 3843-3846.
